# ALTEN: A High‐Fidelity Primary Tissue‐Engineering Platform to Assess Cellular Responses Ex Vivo

**DOI:** 10.1002/advs.202103332

**Published:** 2022-05-25

**Authors:** Andrew M. K. Law, Jiamin Chen, Yolanda Colino‐Sanguino, Laura Rodriguez de la Fuente, Guocheng Fang, Susan M. Grimes, Hongxu Lu, Robert J. Huang, Sarah T. Boyle, Jeron Venhuizen, Lesley Castillo, Javad Tavakoli, Joanna N. Skhinas, Ewan K. A. Millar, Julia Beretov, Fernando J. Rossello, Joanne L. Tipper, Christopher J. Ormandy, Michael S. Samuel, Thomas R. Cox, Luciano Martelotto, Dayong Jin, Fatima Valdes‐Mora, Hanlee P. Ji, David Gallego‐Ortega

**Affiliations:** ^1^ The Kinghorn Cancer Centre Garvan Institute of Medical Research Darlinghurst NSW 2010 Australia; ^2^ Division of Oncology Department of Medicine Stanford University California 94305 USA; ^3^ Cancer Epigenetic Biology and Therapeutics Laboratory Children's Cancer Institute Randwick NSW 2052 Australia; ^4^ School of Women's and Children's Health, Faculty of Medicine University of New South Wales Sydney NSW 2052 Australia; ^5^ Institute for Biomedical Materials and Devices (IBMD) Faculty of Science The University of Technology Sydney Ultimo NSW 2007 Australia; ^6^ Division of Gastroenterology and Hepatology Department of Medicine Stanford University California 94305 USA; ^7^ Centre for Cancer Biology SA Pathology and University of South Australia Adelaide SA 5000 Australia; ^8^ Adelaide Medical School Faculty of Health and Medical Sciences University of Adelaide Adelaide 5000 Australia; ^9^ School of Biomedical Engineering Faculty of Engineering and Information Technology University of Technology Sydney NSW 2007 Australia; ^10^ Department of Anatomical Pathology NSW Health Pathology St George Hospital Kogarah NSW 2217 Australia; ^11^ St George & Sutherland Clinical School UNSW Sydney NSW 2217 Australia; ^12^ Centre for Cancer Research University of Melbourne Parkville VIC 3010 Australia; ^13^ School of Mechanical Engineering University of Leeds LS2 9JT UK; ^14^ Department of Engineering Sciences and Mathematics Luleå University of Technology Luleå 97187 Sweden; ^15^ St. Vincent's Clinical School Faculty of Medicine University of New South Wales Sydney NSW 2010 Australia; ^16^ Single Cell Core Systems Biology Harvard Medical School Harvard University Massachusetts 02115 USA

**Keywords:** alginate, ex vivo drug screening, single‐cell RNAseq, three dimensional culture, tissue microenvironment, whole‐tissue organoids

## Abstract

To fully investigate cellular responses to stimuli and perturbations within tissues, it is essential to replicate the complex molecular interactions within the local microenvironment of cellular niches. Here, the authors introduce Alginate‐based tissue engineering (ALTEN), a biomimetic tissue platform that allows ex vivo analysis of explanted tissue biopsies. This method preserves the original characteristics of the source tissue's cellular milieu, allowing multiple and diverse cell types to be maintained over an extended period of time. As a result, ALTEN enables rapid and faithful characterization of perturbations across specific cell types within a tissue. Importantly, using single‐cell genomics, this approach provides integrated cellular responses at the resolution of individual cells. ALTEN is a powerful tool for the analysis of cellular responses upon exposure to cytotoxic agents and immunomodulators. Additionally, ALTEN's scalability using automated microfluidic devices for tissue encapsulation and subsequent transport, to enable centralized high‐throughput analysis of samples gathered by large‐scale multicenter studies, is shown.

## Graphical abstract

Alginate‐based tissue engineering (ALTEN) enables three‐dimensional (3D) ex vivo culture of explanted tissue biopsies. This method recapitulates the original characteristics of the source tissue's cellular niches with high‐fidelity, including cellular diversity, extracellular matrix (ECM), and their molecular milieu. ALTEN enables rapid and faithful characterization drugs effects in explanted tumoroids as n‐of‐one clinical trials with potential use in personalized medicine.

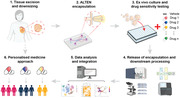

## 1 Introduction

Tissue homeostasis depends on precise cell‐to‐cell communication and assimilation of cell contact, ECM, and paracrine signals. When perturbations are introduced through either small synthetic or biological molecules, each cell's response results from the integration of direct cell intrinsic effects of the perturbation and the signals from the local tissue microenvironment. The lack of reliable experimental systems that recapitulate the normal cellular milieu of tissues is a critical contributing factor to the very low success rate of the discovery of new molecules for biomedical applications (10.4%).^[^ ^1^ ^]^ Improving this performance is dependent on the development of new experimental systems that more accurately model the in vivo responses when screening novel molecules for treatment efficacy.

Currently, most perturbation screens are performed using conventional in vitro methods such as cell lines and more recently organoids; however, these methods have a limited capacity to recapitulate specific complex cellular niches in vivo (recently reviewed in ref. [2]). Cell lines and two‐dimensional (2D) in vitro coculture systems inherently lack heterogeneity and a tissue microenvironment and exhibit poor predictive power of the biological effects of specific stimuli or perturbations.^[^ ^3^ ^]^ Organoids, assembled in vitro from disparate tissue‐derived components, and in particular tumor‐derived organoids, have been increasingly used for assessing drug responses and target validation studies as an in vitro cellular model.^[^ ^3b^ ^]^ However, these rely on the formation of new multicellular entities as a result of the addition of distinct cell types that interact in vitro, forming de novo molecular communications. To generate multilineage epithelial structures, organoid‐based models primarily rely on external supplementation of niche factors due to lack of stromal and immune components. Thus, while superior to 2D cell coculture systems, organoid models are unable to replicate the whole repertoire of cell types, microenvironmental components such as the ECM, and intercellular interactions that are important features to maintain tissue homeostasis.^[^ ^4^ ^]^ Therefore, approaches to culture sections of explanted tissues that maintain normal tissue architecture and survive for lengthy periods has significant appeal. In culturing primary tissue, cell death as high as 90% leads to a dramatic reduction of the cellular diversity and the natural selection of resilient cell types, thereby preventing precise modeling of the native tissue.

Thus, current 3D ex vivo culture methods have a limited capacity to maintain the tissue ecosystem, fail to recapitulate the native ECM components and original cellular configuration, and exhibit extensive cell death. To address these limitations and to faithfully preserve the composition of primary tissues, we developed the ALginate‐based Tissue ENgineering (ALTEN) platform, a versatile and cost‐effective ex vivo tissue system that enables rapid screening and analysis of exogenous molecule perturbation and drug sensitivity testing on native tissue specimens in situ. Unlike in vitro generated multilineage organoids, ALTEN minitissue cultures preserve the original cellular composition of the native tissue, maintain the original 3D features, conserve tissue architecture, replicate cell heterogeneity, and retain cell–cell and cell–ECM communication. Thus ALTEN recapitulates the key properties of the original tissue with high fidelity, offering precise biomimicry.

Alginate is a polysaccharide that has been used for numerous biomedical applications due to its high biocompatibility and adjustable properties, including functionalization with adhesive ligands, proteolytic sites, mechanical strength, and cell affinity. The versatility of alginate as a biomaterial has made it attractive for tissue engineering, wound healing, and drug delivery applications.^[^ ^5^ ^]^ Importantly, alginate does not activate any receptors on mammalian cells, and medical‐grade alginate has low immunogenicity,^[^ ^6^ ^]^ making it an ideal substrate material for maintaining immune cell populations and assessing their ex vivo cellular responses.^[^ ^2^ ^]^ Thus, unlike other materials that may be naturally found in the ECM, alginate does not alter the composition of the original ECM components allowing accurate representation of intact cellular niches.

Alginate forms a biomimetic hydrogel that resembles the ECM under physiological pH and temperature conditions. The tunable stiffness of the encapsulating alginate matrix allows to accurately recapitulate diverse tissue microenvironments for subsequent ex vivo culture and efficient ex vivo manipulation of the whole tissue specimens. The biomimetic properties of the alginate‐hydrogel enable gas exchange and diffusion of nutrients and molecules of varying molecular weight. Alginate hydrogel dissociates with a simple addition of a calcium chelating agent, such as sodium citrate. This property obviates the need of harsh chemicals or mechanical forces to recover cultured tissues and allows multidimensional downstream analysis while minimizing cellular and tissue damage. Cellular response in ALTEN can be measured using conventional cell biology techniques such as microscopy, flow cytometry, or histology, but also highly parallel “omics” approaches such as single‐cell RNA sequencing (scRNA‐seq).

Herein, we demonstrate the capacity of ALTEN to preserve tissue characteristics and demonstrate its utility as a method for using primary tumor biopsies for molecular perturbation and drug screens. We verified ALTEN's capacity across a series of metrics: the extent of cellular preservation the tissue complexity of tumors under steady‐state culture conditions and the consequences of specific perturbations that targeted epithelial cells with cytotoxic drugs and infiltrating immune cells with immunomodulators. To assess these metrics, we used a combination of flow cytometry, microscopy, and high‐resolution single‐cell genomics (scRNA‐seq). We demonstrate the advantage of ALTEN as a tissue preservation method and develop a microfluidic device for high‐throughput applications. These comparisons included conducting ex vivo assay of drug sensitivity, functional characterization of pharmacological heterogeneity and biomarker discovery/validation.

## 2 Results

### 2.1 ALTEN Preserves Tissue Architecture and Cell Viability Ex Vivo

**Figure 1:**
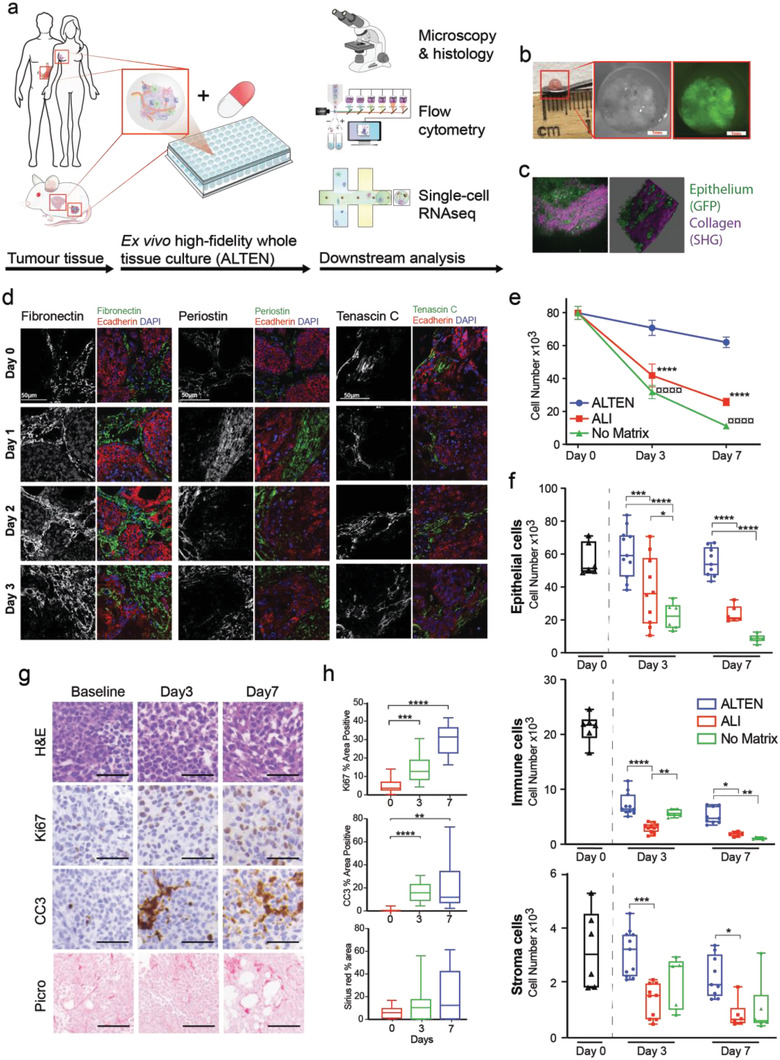
ALTEN preserves tissue architecture and cell viability ex vivo. a) Schematic representation of the ALTEN methodology. b) Macroscopic and stereo microscopy pictures of a MMTV‐PyMT/GFP+ ALTEN‐engineered tumoroid, Green: GFP expression restricted to mammary epithelial cells. c) Multiphoton microscopy image and 3D projection of an encapsulated MMVT‐PyMT/GFP tumor. Green: GFP expression restricted to mammary epithelial cells, purple: second harmonic generation (SHG) signal from collagen fibers within the tumor extracellular matrix. d) Immunofluorescent analysis of the main ECM components, fibronectin, periostin, and tenascin C (greyscale and green) in ALTEN encapsulated MMTV‐PyMT tumors for 1, 2, and 3 days. Epithelial cells are stained with E‐cadherin (red) and nuclei counterstained with DAPI (blue). e) Comparison of live cell number assayed by flow cytometry from cell suspensions of 4T1.2 mammary carcinomas (equivalent volume) cultured in ALTEN (blue line), ALI (red line), and as a naked explant (green line) compared to the cell number yield of the fresh tissue (baseline, time = 0) (*n* ≥ 5). f) Flow cytometric analysis of live cell number as in (e) but divided by main lineage, (4T1.2 cancer cells are defined by expression of mCherry, immune cells are defined by CD45, and stromal cells are defined as double negative). g) Representative histology images of ALTEN‐engineered 4T1.2 tumoroids stained by H&E, Picrosirius red (collagen), or immunolabeled for cleaved caspase 3 (CC3) and Ki67 antibodies. h) Quantification of the area stained in the histology images (*n* ≥ 5). Data are represented as mean ± SEM. *p*‐values are calculated using one‐way ANOVA testing followed by Dunnett's multiple comparison test, *p < 0.05, **p < 0.01, ***p < 0.001, ****p < 0.0001.

We tested the capacity of ALTEN to preserve the tissue architecture and a variety of cell types across a wide variety of organs, of both normal (mammary tissue) and neoplastic origin (breast, pancreatic and colorectal biopsies, and mammary tumors) and of murine and human origin. In all cases, freshly collected tissue was minced into small pieces and directly encapsulated in an alginate‐based hydrogel as described in the Experimental Section (**Figure** **1**a). For example, pieces of murine mammary tumors with a general diameter of 0.5–1 mm^3^ were encapsulated in alginate, thus forming intact whole‐tissue tumoroids (Figure 1b).

Alginate is a transparent material that allows high‐end image analysis using multiphoton microscopy of the embedded tissue. ECM components and the overall 3D architecture of the tumor were maintained in ALTEN (Figure 1c,d and Figure S1a, Supporting Information). As alginate is not a component of mammalian tissue, all these ECM components were originally present in the embedded specimens. ALTEN tumoroids from mouse mammary carcinomas (4T1.2) were cultured for 3 and 7 days. As a comparison, tumor pieces from 4T1.2 were also cultured without any matrix support and in air–liquid interface (ALI) using a gelatine sponge platform, another technique used for ex vivo culturing of tissue.^[^ ^7^ ^]^ Afterward, cell viability was assayed by flow cytometry (Figure S1b, Supporting Information). The overall cell recovery after tissue dissociation of ALTEN whole‐tissue tumoroids was not significantly different at day 3 compared to the original baseline (day 0) sample, and showed a nonsignificant trend toward decrease at day 7. However, in ALI culture conditions and tissues cultured with no matrix, there was significantly higher cell death from day 3 onwards, depleting cells by 70% in ALI and 85% with no matrix by day 7 (Figure 1e and Figure S1c, Supporting Information). These results also suggest that the artificial ECM provided by alginate acts as a physical barrier to preserve tissue integrity and prevent tumor cell scaping to the plate plastic.

Compared to unencapsulated or ALI tissue culture conditions, ALTEN encapsulation more effectively preserved epithelial and fibroblast lineages (Figure 1f and Figure S1d, Supporting Information), and only a significant reduction on cell maintenance occurred among immune cells (Figure 1f and Figure S1d, Supporting Information). Nevertheless, in all cases, ALTEN culture outperformed the other tissue culture techniques (Figure 1f). This initial loss of immune cells was presumably due to the intrinsic short‐lives nature of some of these cell species in the absence of specific, likely systemic, immune stimuli. Additionally, a live cell tracer analysis showed that cancer cells continued proliferating during the period of ex vivo culture (Figure S1e, Supporting Information). Similar results were obtained with the highly heterogeneous MMTV‐PyMT tumors (Figure S2, Supporting Information). While PyMT tumoroid integrity was generally maintained during ex vivo culture in all techniques assayed, cell viability was significantly higher in ALTEN compared with ALI and unencapsulated tissue (Figure S2a,b, Supporting Information), maintained sustained cancer cell proliferation, and a close similarity to the original tumor cell composition (Figure S2c, Supporting Information).

Cell proliferation and apoptosis were further analyzed by Ki67 and cleaved caspase 3 (CC3) immunohistochemistry staining on sections of the cultured ALTEN tumoroids and overall collagen content by Picrosirius red staining (Figure 1g,h). Ki67 labeling supported the cell trace data, showing active proliferation. However, although the overall viability of the cells was not significantly altered by ALTEN tumoroid culture, we identified regions of the tissue undergoing apoptosis (Figure 1g) reflecting underlying tissue heterogeneity and potentially accounting the loss of immune cells. Overall collagen content remained largely stable (Figure 1g,h). Finally, we analyzed key mechanical properties of large (>5 mm) cultured MMTV‐PyMT tumor pieces. Cultured tissues showed a higher Young's modulus, resilience, and maximum resistance compared with the fresh tissue, however, ALTEN encapsulated and nonencapsulated tumors exhibited comparable mechanical parameters (Figure 1f). These results suggest that as part of the adaptation to the culture conditions, ex vivo cultured tissues acquire increased resistance to mechanical stress.

Similarly, ALTEN was used to successfully culture tissue from a variety of models including the 67NR syngeneic breast cancer model (Figure S3a–g, Supporting Information); normal mammary tissue (Figure S4a,b, Supporting Information), as well as preserving an array of human cancer tissues, including breast (Figure S4c, Supporting Information), pancreatic, and colorectal cancer (Figure S4d, Supporting Information).

Tissues have varying degrees of cellular diversity, and in particular tumors present an extensive degree of intratumor heterogeneity,^[^ ^8^ ^]^ encompassing cancer epithelial clones associated to local microenvironments composed of diverse immune and stromal cell types.^[^ ^9^ ^]^ Using flow cytometry, we confirmed that the cellular composition of ALTEN‐encapsulated tissue recapitulated the heterogeneity of baseline tissue from its original source (Figure S5, Supporting Information). For example, 4T1.2 tumors showed high levels of heterogeneity in both baseline and ALTEN‐cultured conditions while the heterogeneity was not as extensive in fresh and ALTEN‐cultured lung tissue of the same mouse model, suggesting that ALTEN is amenable to preserve the original cell diversity of tissues of differing complexities.

### 2.2 ALTEN Is a High‐Fidelity Ex Vivo Platform for Whole‐Tissue Drug Perturbation Analysis

**Figure 2:**
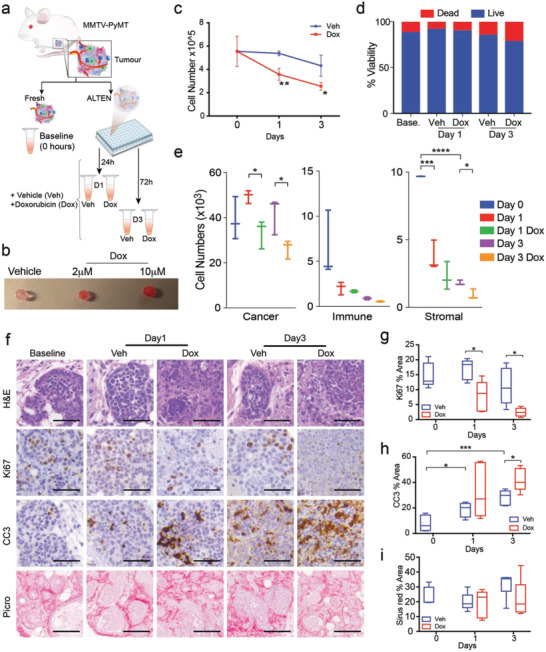
ALTEN is a high‐fidelity ex vivo platform for whole‐tissue drug perturbation analysis. a) Schematic representation of the experimental design. b) Macroscopic images of ALTEN hydrogels incubated 24 h with doxorubicin (red) at the indicated dose. c,d) Cell number and cell viability assayed by flow cytometry (DAPI) from cell suspensions of fresh (baseline, time = 0) or ALTEN‐cultured MMTV‐PyMT mammary carcinomas. e) Number of live cells obtained on the tissues assayed in panel (c) and divided by major lineage (PyMT cancer cells are defined by expression of EpCAM, immune cells are defined by CD45, and stromal cells are defined as double negative). f) Representative histology images of ALTEN‐engineered PyMT tumoroids stained by H&E, Picrosirius red (collagen), or immunolabeled for cleaved caspase 3 (CC3) and Ki67 antibodies. g–i) Quantification of the area stained in the histology images in response to doxorubicin (*n* ≥ 5). All captions: Veh = vehicle, DOX = doxorubicin. Data are represented as mean ± SEM. *p*‐values are calculated using one‐way ANOVA testing followed by Dunnett's multiple comparison test, *p < 0.05, **p < 0.01, ***p < 0.001, ****p < 0.0001.

We characterized the utility of the ALTEN platform for assaying drug sensitivity in whole tumor specimens. These experiments took advantage of the MMTV‐PyMT mouse model that develops spontaneous mammary carcinomas that closely resembles the histopathology of human breast cancers.^[^ ^10^ ^]^ To characterize the drug response of these tumors, ALTEN‐engineered MMTV‐PyMT tumoroids were cultured in the presence of a low dosage of doxorubicin (DOX) or vehicle for 1 and 3 days (**Figure** **2**a) and DOX permeation to the hydrogel was assessed (Figure 2b). Similar to the 4T1.2 and 67NR models, cell viability and tissue architecture in spontaneous MMTV‐PyMT tumors were preserved (Figure 2c–e). As expected, DOX‐treated samples had lower cell proliferation and elevated apoptosis as assayed by Ki67 and CC3 immunostaining, respectively, relative to vehicle controls at each time‐point. Collagen architecture was maintained as shown by Picrosirius red staining (Figure 2f–i).

Subsequently, we analyzed 25000 tumor cells using scRNA‐seq to investigate molecular responses to ALTEN ex vivo tissue culture, and the response to DOX, at single‐cell resolution. To ensure an appropriate level of sampling and capture the whole heterogeneity, this analysis was based on 96 hydrogels randomly plated and distributed between groups and days. After exploring different integration approaches (see Supporting Information for detailed description) batch effects between experiments captured in different days (baseline‐batch 1, day 1‐batch 2, day 3‐batch 3) were corrected using the anchor‐based integration method from Seurat V3^[^ ^11^ ^]^ (Figure S6, Supporting Information). Based on their transcriptional characteristics, ALTEN‐cultured cells had similar quality control metrics and cell numbers compared to the freshly prepared scRNA‐seq capture (baseline – 0h) of the same tumors sample (Figure S7a–c, Supporting Information). A dimensional reduction UMAP visualization showed that cells analyzed from the baseline sample greatly overlapped with samples cultured in ALTEN for 1 and 3 days (Figure S7d,e, Supporting Information). We subsequently interrogated this dataset following the workflow depicted in Figure S7f, Supporting Information.

**Figure 3:**
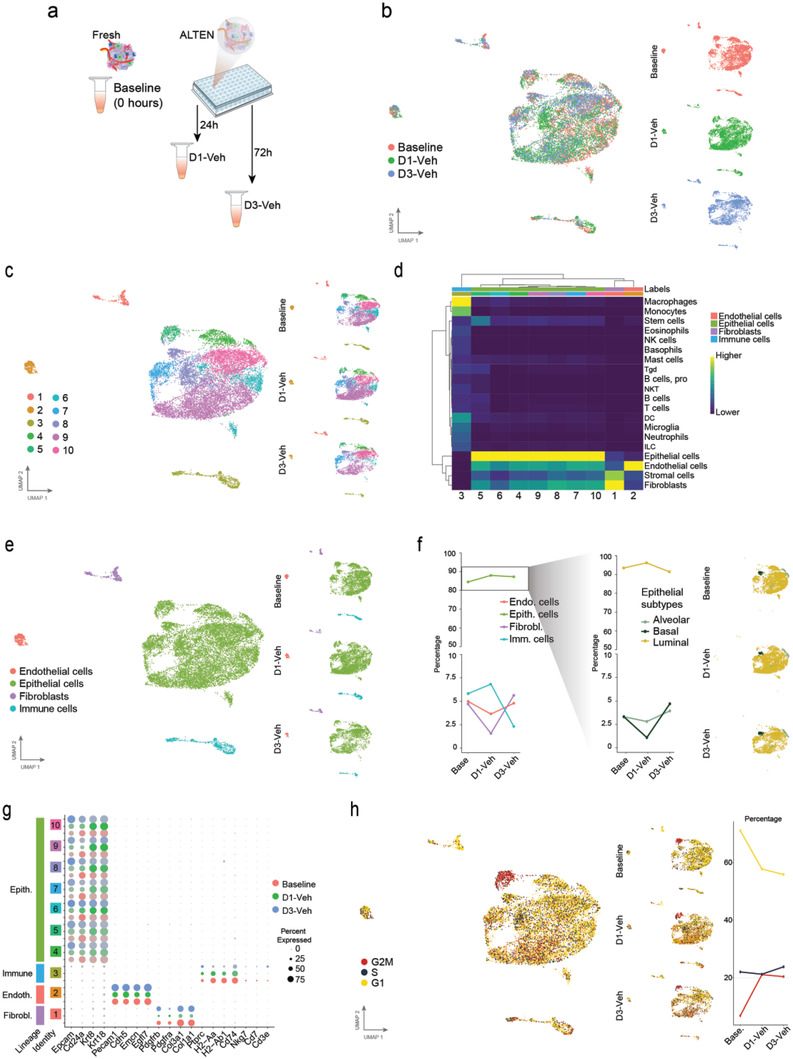
ALTEN preserves tumor complexity and cell diversity measured by scRNAseq. a) Schematic representation of the experimental design. b) UMAP projection of ALTEN‐engineered tumoroids cultured 1 (5474 cells) and 3 days (3156 cells) compared with fresh tissue (baseline, 5048 cells) normalized using SCTransform function and anchor‐based integration from Seurat. c) UMAP projection showing unsupervised clustering overall (left) and split by time point (right). d) Heatmap showing the single R score of each unsupervised cluster against the mouse cell type reference signature generated by the Immunologic Genome Project.^[^ ^12a,b^ ^]^ e) UMAP projection showing cell lineage based on single R analysis overall (left) and split by time point (right). f) Line plot showing the percentage of cells from each lineage in the baseline condition and after 1 day or 3 days of culture in ALTEN (left). The right panel shows the percentage of cells in each epithelial cell type per culture condition and the epithelial subtypes plotted in the epithelial UMAP. g) Dot plot showing the comparison between culture conditions in the level of expression of top cell lineage markers in each cell cluster. h) UMAP projection showing cell cycle phase (left) and the percentage of cells from each phase at each culture timepoint (right).

We compared the impact of ALTEN ex vivo culturing on the cell diversity and molecular repertoire of the tumoroids compared with the baseline fresh sample at the time of acquisition (**Figure** **3**a). Cells analyzed from the baseline uncultured sample clustered together with the vehicle‐treated samples at days 1 and 3 (Figure 3b). Importantly, intrinsic cell diversity was also preserved from the original tumor tissue at the time of acquisition, identifying ten distinct cell clusters using unsupervised analysis, which were all preserved at the original tissue (baseline) and ALTEN culture conditions (Figure 3c). The proportion of cancer cell clones varied across days, presumably due to the intense heterogeneity of this cellular compartment, which was consistent with our previous observations using flow cytometry (Figure S5, Supporting Information), and without any drop out on the presence of any cluster during the cultured period. We then used established gene signatures generated by the Immunologic Genome Project^[^ ^12^ ^]^ and canonical markers for PyMT tumors^[^ ^13^ ^]^ to annotate the cell identity of each cell cluster (Figure S3d, Supporting Information). We identified immune cells, fibroblast/stromal cells, endothelium, and a diverse range of epithelial cells; and consistent with previous scRNA‐seq analyses of tumors from the MMTV‐PyMT model^[^ ^13^ ^]^ (Figure 3e). Consistent with the flow cytometry data from Figure 2e, the ALTEN culturing conditions conserved all of the major tumor cell lineages over a 3‐day period. Cancer cells made up the majority ranging from 85% to 88% of recovered cells; followed by stromal cells, 5.5–10.1% (3.7–5% of endothelial cells and 1.8–5.1% of fibroblasts); and immune cells, 2.5–6.9% of recovered cells (Figure 3f, left panel, and Figure S8a,b, Supporting Information). Furthermore, cancer heterogeneity including the three main epithelial cell lineages of this model, alveolar, basal, and luminal, were also maintained, with no significant loss of specific populations (Figure 3f, right panel, and Figure S8c–f, Supporting Information).

ALTEN culture conditions preserved the integrity of the biological features across all the cellular compartments (Figure 3g). Cancer cells were characterized by strong expression of canonical mammary epithelial cell markers, *Epcam* and *Cd24*, and including luminal markers Krt8 and *Krt18*, consistent with the luminal progenitor nature of MMTV‐PyMT tumors. Endothelial cells strongly expressed Pecam1 (CD31), Cdh5, Emcn, and Egfl7. Fibroblasts were characterized by *Pdgfra* and *Pdgfrb* and the expression of fibrillar collagen subunits *Col3* and *Col1*. Immune cells were characterized by the expression of *Ptprc* (CD45), histocompatibility‐associated molecules (*H2‐Aa*, *H2‐Ab* and *Cd74*) and other minor cell populations characterized by *Nkg7* expression (NK and T CD8 cells) and *Cd3* and *Cd7* expression (T cell markers). ALTEN‐engineered cells continued proliferating as shown by scRNAseq cell cycle signatures (Figure 3h). In fact, the proportion of cells in G2M was higher in ALTEN‐cultured cells than at baseline. These data might indicate the conservation of viability of all cell populations from day 1 and 3, potentially due to improved access to nutrients within the culture conditions.

### 2.3 Molecular Classification of Epithelial Cell Sensitivity to Doxorubicin

**Figure 4:**
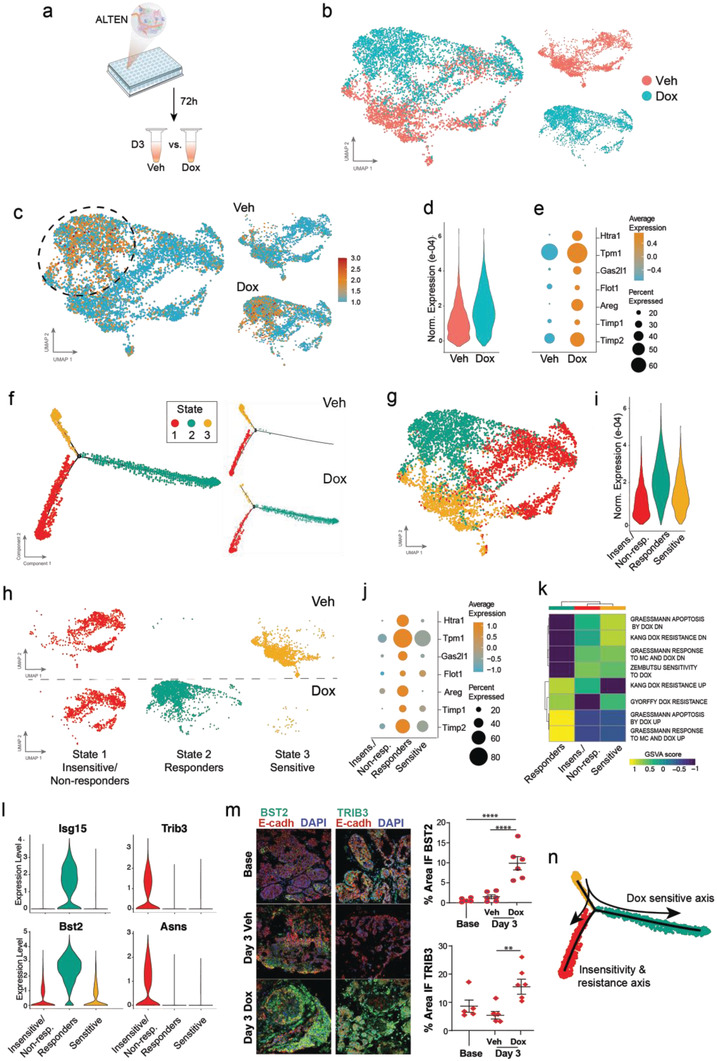
Detecting the molecular response of Doxorubicin treatment in ALTEN‐tumoroids. a) Schematic representation of the experimental design. b) UMAP projection of epithelial cells cultured in the ALTEN system for 72 h with doxorubicin (DOX, 3777 cells) or vehicle (Veh, 2843 cells) normalized using SCTransform function from Seurat. c) UMAP visualization showing the metagene signature of doxorubicin response (see Table 1) overall (left) and split based on condition (right). d) Violin plot showing the normalized expression of the metagene signature of doxorubicin response in vehicle and doxorubicin treated cells. e) Dot plot showing the percentage of expressing cells and average expression of seven genes from the doxorubicin response signature in untreated (Veh) and treated (DOX) cells. f) Trajectory analysis of cancer epithelial cells based on differential analysis between the vehicle‐ and DOX‐treated cells using the DDRTree method in Monocle2 and colored by states. Right panels show the distribution of the cell states by condition. g) Projection of the states defined by pseudotime analysis into the UMAP coordinates. h) Projection of the states defined by pseudotime analysis into the UMAP coordinates per trajectory state and condition. i) Violin plot showing the normalized expression of the metagene signature for doxorubicin response of each state (1: insensitive/Resistant, 2: DOX committed, 3: DOX sensitive). j) Dot plot showing the percentage of expressing cells and average expression of seven genes from the doxorubicin response signature in each DOX‐dependent state. k) Enrichment analysis (GSVA score) for doxorubicin response gene sets. l) violin plot for marker genes from gene sets in panel (k). m) Immunofluorescence of Bst2, Trib3, and E‐Cadh of PyMT‐derived ALTEN tumoroids at baseline (*t* = 0) and after 3 days in culture exposed to doxorubicin (DOX) a vehicle control (Veh). n) Summary of the doxorubicin response axis at the single‐cell resolution. Data are represented as mean ± SEM, *n* = 5. *p*‐values are calculated using one‐way ANOVA testing followed by Dunnett's multiple comparison test, **p < 0.01, ****p < 0.0001.

We then analyzed the early transcriptomic effects of DOX in the ALTEN cultured tumoroids (**Figure** **4**a and Figure S7e,f, Supporting Information). To determine the DOX effects on the ALTEN tumoroids, we first considered the cancer epithelial cells from the day 3 vehicle‐ and DOX‐treated ALTEN samples. UMAP visualization revealed a strong transcriptional shift in response to DOX in epithelial cells (Figure 4b). A DOX response signature^[^ ^14^ ^]^ (**Table** **1**) identified cells that occupied a different transcriptional space compared to the vehicle‐treated cells (Figure 4c), and this signature was clearly enhanced in the DOX‐treated sample (Figure 4d). Specifically, we identified a higher proportion of cells enriched for transcriptional components involved in DOX action, as well as higher expression of these specific genes per cell (Figure 4e). For example, DOX‐treated cells presented de novo expression of *Htra1*, *Gas2l1*, or *Areg*; genes associated with cellular response to DOX and known to modulate chemotherapy‐induced cytotoxicity.^[^ ^15^ ^]^

**Table 1:** Doxorubicin gene signature

	Gene symbol	Rank
1	Htra1	Top differential
2	Timp1	Top differential
3	Casp1	Top differential
4	Timp2	Top differential
5	Gas2l1	Top differential
6	Flot1	Top differential
7	Areg	Top differential
8	Brap	Top differential
9	Exoc4	Top differential
10	Tpm1	Top differential
11	Smarcd2	Top differential
12	Phlda3	Top differential
13	Notch1	Top differential
14	Mtss1	
15	Clptm1	
16	Ifi27	
17	Nampt	
18	Cdkn1a	
19	Cpne3	
20	Apobec3	
21	Smyd2	
22	Ap3m1	
23	Vapb	
24	Tspo	
25	Nckap1	
26	Myc	
27	Stxbp2	
28	Cope	
29	Rab5c	
30	Rab1b	
31	Rin2	
32	Mfsd10	
33	Tm9sf4	
34	Trappc1	
35	Kifc2	
36	Mark2	
37	Dctn2	
38	Arpc1a	
39	Actr1b	
40	Pawr	
41	Tdp2	
42	Gmfc	
43	Cetn3	
44	Pcm1	
45	Psmc6	
46	Psmd3	
47	Gtf2e2	
48	Cald1	
49	Lima1	
50	Emp1	
51	Clstn1	
52	Slc38a2	

We next used unsupervised pseudotime trajectory alignment based on differential analysis between vehicle‐ and DOX‐treated samples to analyze the sequential relationship of cancer cells in response to DOX. Overall, we identified three distinct states (Figure 4f). Vehicle‐treated cells were clearly enriched for State 3, while DOX treated cells were enriched for State 2. The proportion of cells in State 1 was comparable between conditions. The distribution of the identified cellular states within the UMAP‐generated coordinates confirmed that State 2 strongly correlated with DOX responsive cells while State 3 was localized in a transcriptional space unique to cells only present in the vehicle sample (Figure 4g,h). These results suggest a State 3 (“DOX sensitive”)‐to‐State 2 (“DOX responders”) transition over a DOX sensitivity axis. The DOX responder cells accumulated most of the expression of the transcriptional components of the DOX response signature (Figure 4i,j). State 1 remained unchanged upon DOX exposure, representing cells that were either insensitive or unresponsive to the effects of the drug or potentially cells that presented innate resistance. This cancer cell classification according to their sensitivity to DOX was also confirmed with published signatures of DOX action in breast cancer^[^ ^14^, ^16^ ^]^ (Figure 4k). Responder cells showed the greatest correlation with signatures of associated with DOX response (“Garessmann response to MC and DOX up”) and apoptosis (“Garessmann apoptosis by DOX up”), thus supporting the idea that these cells were on trajectory to cell death due to the effects of DOX. Insensitive/nonresponder cells however, showed the opposite trend with the highest expression of genes that anticorrelate with response to DOX (“Garessmann response to MC and DOX down”). Accordingly, sensitive cells prior to DOX exposure showed the highest correlation with genes involved in DOX sensitivity (“Zembutsu sensitivity to doxorubicin”). Genes differentially expressed in the “responder” cell state constitute potential biomarkers to identify the pharmacological action of DOX and individualized chemotherapy response in patient's tumor biopsies. For example, *Isg15* and *Bst2* encode proteins that participate in chemosensitivity associated to DOX; and *Trib3* and *Asns*, which have been previously associated with molecular pathways of DOX resistance (Figure 4l). Immunofluorescence analysis of BST2 and TRIB3 in ALTEN tumoroids confirmed a direct correlation between identified transcriptional candidates and their value as potential protein‐based biomarkers to assess DOX responses (Figure 4m). As shown in Figure S5d,q–t, Supporting Information, the candidates *Isg15* and *Bst2* were robustly identified regardless the integration method. A list of the top candidates associated with DOX response and DOX insensitivity can be found in Table 1. A summary of the cellular trajectories is depicted in Figure 4n.

Cells treated with DOX for 1 day (Figure S9a, Supporting Information) followed a similar trend to the day 3 treatment. The effects of DOX exposure for 1 day also showed a transcriptional difference between vehicle‐ and DOX‐treated samples but less pronounced than that to day 3, highlighted by a divergence of a subset of cells in the DOX‐treated sample (Figure S9b, Supporting Information), and consistent with the gradual emergence of a DOX response signature (Figure S9c–e, Supporting Information).

Finally, to illustrate the value of ALTEN as a testing platform for personalized medicine, we evaluated ALTEN's capacity to profile the sensitivity of tumors to multiple drugs. MMTV‐PyMT mammary tumors' pieces were encapsulated and cultured in a multiwell plate and exposed to several drugs that targeted diverse molecular targets apart from DOX, including the Bcl‐2 inhibitor Navitoclax (ABT‐263), the actin polymerization inhibitor latrunculin A, and the MLC‐1 inhibitor S63845 or a vehicle control (Figure S10a, Supporting Information). Analysis of cell viability in response to these treatments showed differential responses of the MMTV‐PyMT tumors, which was more pronounced with higher drug concentrations (Figure S10b, Supporting Information).

Taken together, our single‐cell molecular analysis demonstrates that ALTEN ex vivo culturing is a bona fide and reliable model for the faithful preservation of the cellular diversity and molecular repertoire of tumor specimens, suitable for the generation of high‐resolution vignettes of chemotherapeutic responses ex vivo; and subsequently, useful as a n‐of‐one clinical trials and biomarker discovery tool.

### 2.4 Ex Vivo Immunomodulation of Tumor Infiltrating Immune Cells in the Tissue Microenvironment

**Figure 5:**
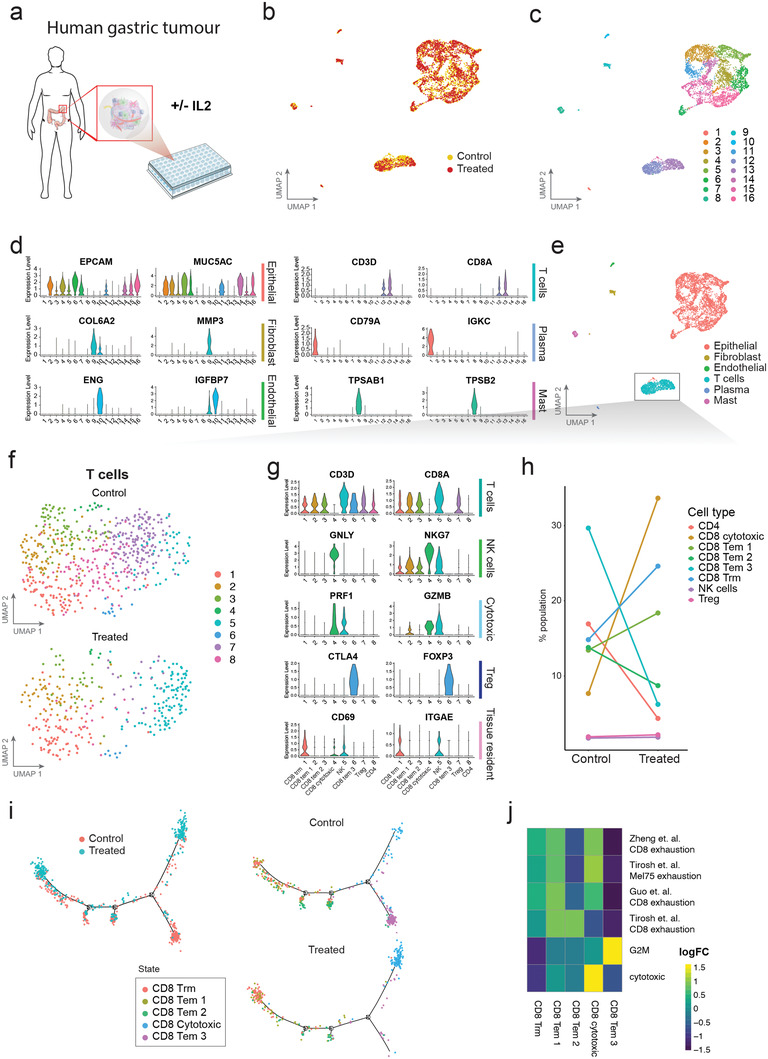
Immunomodulation of gastric intestinal metaplasia (GIM) infiltrated immune cells ex vivo. a) Schematic representation of experimental workflow. b) UMAP projection of human GIM tissue cultured in the ALTEN system for 24 h and colored based on condition, control (yellow, 2754 cells) or IL2 treated (red, 2501 cells). c) UMAP projections colored based on k‐nearest neighbor clustering. d) Violin plots showing normalized expression of marker genes for epithelial, fibroblast, endothelial, T, Plasma, and Mast cells. e) UMAP projections colored based on cell lineages. T cells are indicated by the boxed area. f) UMAP projection of T cells colored based on k‐nearest neighbor clustering and split by condition (control, 573 cells and IL2, 321 cells). g) Violin plot showing normalized expression of marker genes for T cells, natural killer (NK) cells, cytotoxic T cells, regulatory T cells (Treg), and tissue resident memory T cells (TRM). h) Line plot showing percent of cells from each cell type per condition. i) Trajectory analysis of the T cells based on differential analysis between the control and IL2 treated cells using the DDRTree method in Monocle2 and colored by condition (left) or by cell type and split by condition (right). j) Enrichment analysis (GSVA score) for CD8 T signature gene‐sets.^[^ ^19^ ^]^

We next investigated whether ALTEN enables the study of pharmacological effects on specific immune cell populations present in primary human tissues. Specifically, we assessed the effects of immunomodulation of T cells within the human tissue microenvironment. First, we encapsulated tissue biopsies from a patient with gastric intestinal metaplasia, a precursor to gastric carcinoma. ALTEN‐engineered tumoroids were randomized, exposed to interleukin‐2 (IL2) or vehicle control for 24 h and were subsequently analyzed by scRNAseq (**Figure** **5**a). IL2, a well‐characterized pleiotropic cytokine, plays crucial roles in immune response, and is one of the early immunotherapy drugs approved by the U.S. Food and Drug Administration for metastatic cancer.^[^ ^17^ ^]^ A recent study revealed that IL2 promotes a tumor‐infiltrating CD8+ T‐cell response.^[^ ^18^ ^]^

Similar to the mouse model described above, single cell transcriptome data demonstrated that ALTEN preserved the cell diversity of human gastric biopsies, including multiple epithelial cell types, fibroblasts, endothelial cells and a variety of immune cells (Figure 5b,d and Figure S11a–c, Supporting Information) in both vehicle control and IL2‐ treated samples. To characterize the effects of IL‐2 stimulation, we selected T cells for downstream analysis (Figure 5e). In total, we identified eight cell clusters, including five T CD8 subtypes: resident memory (Trm), type 1 effector memory (Tem), type 2 Tem, type 3 Tem, and cytotoxic; two T CD4 subtypes (Treg and CD4); and one NK cell type that had been grouped with T cells during previous integration analysis (Figure 5f). Specifically, IL2 treatment substantially increased the proportion of cytotoxic T CD8 cells from 7.6% to 33.6% in the T cell composition (Figure 5g,h). Furthermore, IL2 increased the proportion of cytotoxic T CD8 cells expressing higher levels of genes encoding cytotoxic proteins, that is, GZMB and PRF1 (Figure S11d, Supporting Information). Unsupervised pseudotime cell trajectory analysis revealed that IL2‐induced cytotoxic T CD8 cells are derived from type 3 effector memory T cells (Figure 5i). Gene‐set variation analysis (GSVA), based on known T CD8 signature gene‐sets,^[^ ^19^ ^]^ confirmed the cytotoxic gene signature in the cytotoxic T CD8 cells and revealed that type 3 effector memory T cells are enriched with G2M genes (Figure 5j). In addition, we did not notice any significant effects of IL2 on other stromal cell populations, including plasma (Figure S12a, Supporting Information), mast (Figure S12b,d, Supporting Information), and fibroblast cells (Figure S12c,e, Supporting Information). To confirm these results, we performed a similar analysis using a human breast cancer specimen, cut into pieces, engineered in ALTEN and randomized prior to IL2 or vehicle exposure. After 24 h of incubation, T cells were analyzed using scRNA‐seq. 11 different clusters of T cells were defined (Figure S13a,b, Supporting Information), and in agreement with our previous results, cytotoxic T cell and CD4+ activated cell clusters were greatly enriched in the IL2 group relative to the vehicle control (Figure S13c,d, Supporting Information). Unsupervised trajectory analysis of T CD8 cells revealed a clear distinct fate for the cytotoxic T cell clusters (States 3, 4, and 5) in response to IL2 (Figure S13e,f, Supporting Information). These results were further validated using the GSVA with the T cell signatures (Figure S13g, Supporting Information).

Taken together, our data using the ALTEN platform demonstrated that IL2 primarily induced CD8 T cells into cytotoxic cells in the tumor microenvironment at 24 h using the ALTEN platform, recapitulating the effects of IL2 observed in vivo recently.^[^ ^18^ ^]^ These results suggest that ALTEN provides an excellent novel platform for ex vivo immunomodulation studies.

### 2.5 Upscaling ALTEN for Multicenter High‐Throughput Processing Studies

**Figure 6:**
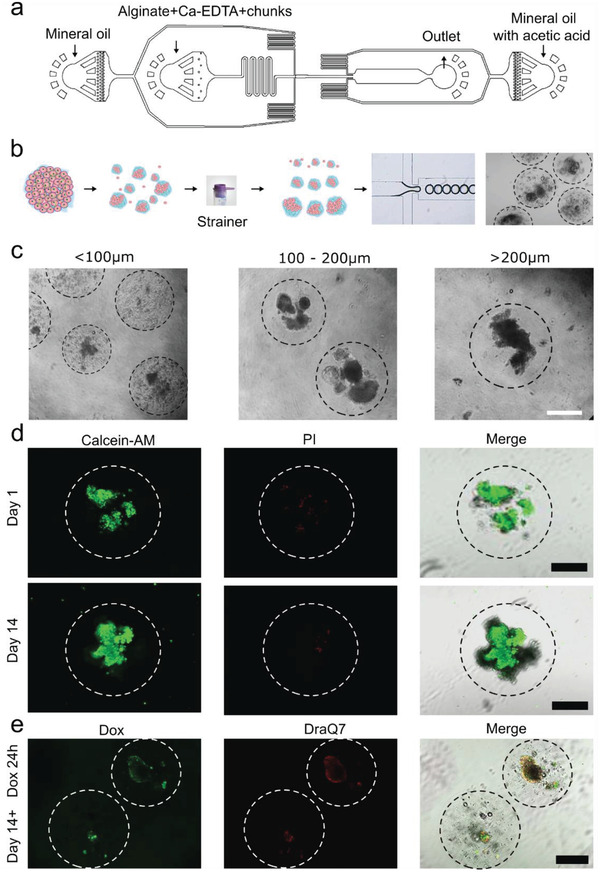
Implementation of the ALTEN microfluidic device. a) Blueprint of the microfluidic chip. b) Schematic representation of the process of automated the tissue ALTEN‐engineering. c) Transmitted light microscopy pictures of ALTEN‐engineered tumoroids of different sizes generated by scalable‐size ALTEN devices. d) Fluorescent microscopy images of 200 µm ALTEN‐engineered tumoroids generated using the ALTEN device stained with calcein AM (green) highlighting live cells and propidium iodide (PI, red) revealing dead cells after 1 and 14 days of culture. e) Doxorubicin (DOX) exposure for 24 h on 14‐day cultured ALTEN‐engineered tumoroids, DOX (green), and DraQ7 (Red) (Scale bars = 200 µm).

To facilitate tissue processing for large‐scale studies, we designed an automated microfluidic ALTEN encapsulation device (**Figure** **6**a). Microfluidic devices have been traditionally used to streamline the production of hydrogels of cell suspensions enabling high throughput screening assays. We designed a microfluidic device capable of encapsulating tissue fragments of different sizes. This device allows for precise control of the size and shape of the ALTEN hydrogels and can produce a monodisperse solution of hundreds of hydrogels in just a few minutes (Figure 6b). This ALTEN microfluidic chip increase reproducibility, tissue sampling capacity, and improve the processing ability of small amounts of tissue with less wasted cellular material. We used the ALTEN microfluidic device to encapsulate tissue pieces of different sizes, ranging from 100 to 500 µm (Figure 6c), indicating its scalability for processing tissues of different sizes.

200 µm‐size ALTEN hydrogels encapsulating PyMT tumors' sections from the ALTEN device showed high viability during long‐term (14 days) culture (Figure 6d). At day 14, ALTEN‐engineered PyMT tumoroids were exposed to DOX for 24 h, similarly to the results shown above, DOX permeated through the alginate matrix and reached the tumoroid cells (Figure 6e). We further demonstrated the versatility of the ALTEN device for culturing lung and mammary tissue from a healthy mouse donor for 14 days (Figure S14, Supporting Information).

A second necessary component to facilitate large‐scale studies from different sites is normalization. We tested ALTEN‘s ability to maintain the fidelity of 3D tissue architecture and viability for transportation of tissue biopsies. MMTV‐PyMT tumor pieces were embedded in ALTEN and sent interstate (>870 km), from Sydney (Syd) to Melbourne (Mel) at room temperature using a regular overnight courier service—disrupting the cold chain for conventional tissue transport. ALTEN hydrogels were received the next day (after ≈20h) and processed normally. Upon digestion, transported ALTEN tumor cell viability was 80% as assayed by cell counting using trypan blue exclusion. The scRNAseq analysis of the transported sample (Mel) produced a similar quality output to freshly processed tissue processed (Syd), including comparable mitochondrial‐to‐nuclear gene rates (Figure S15a, Supporting Information). UMAP analysis revealed a great extent of clustering overlap and lineage identification between the two samples (Figure S15b, Supporting Information) and recapitulated the cell diversity observed in the baseline fresh sample (Syd) (Figure S15c–e, Supporting Information). These results are consistent with those previously obtained in the ALTEN culture condition assessment shown in Figure 3. Similarly, ALTEN‐cultured tumoroids showed an increase in cells with G2M signature (Figure S15f, Supporting Information).

Thus, ALTEN is a versatile and scalable method that enables the transport and high‐fidelity preservation of cell viability and diversity and overall tissue architecture, opening the door to standardized studies for multicenter initiatives such as large consortiums.

## 3 Conclusions

Alginate hydrogels have been extensively used in biomedical applications due to their biocompatibility, low immunogenicity, and ease of gelation. These biomimetic matrices have been widely used as scaffolds to recreate 3D cellular structures, enabling in vitro studies of cell interactions with ECM components.^[^ ^20^ ^]^ Here we have harnessed the properties of alginate matrices to preserve whole tissue pieces for ex vivo culture. ALTEN creates a permeable alginate sphere that preserves the original tissue architecture, cellular diversity, and ECM. Specifically, this approach maintains an intact tissue microenvironment. Unlike other methods for whole‐tissue culture such as ALI, this platform greatly preserves cell viability of multiple lineages, maintaining cellular niches, and appropriate heterotypic cross‐talk between epithelial cells and immune/stromal cells within their ECM. Sustaining these complex cellular interactions is a critical component for studying transcriptional responses influenced by complex tissue architecture at the single‐cell level. Recently, the Human Cell Atlas Consortium^[^ ^21^ ^]^ has made major advances in characterizing cell types and lineages in their physiological state. The next logical step is to study the function of these cell types in their niche and in the context of chemical or biological perturbations. The ability of ALTEN to preserve the intact tumor ecosystem cost‐effectively and rapidly presents a unique opportunity to perform perturbation studies at single‐cell resolution capturing the full repertoire of cellular interactions of the different lineages within a tissue.

In this study, we highlight the utility of the ALTEN platform to investigate the effects of cell perturbation using tumors, a highly heterogeneous tissue, in response to a cytotoxic drug. We used an ALTEN‐engineered mammary tumor model coupled with scRNAseq analysis to reveal heterogeneous cellular responses to the topoisomerase II inhibitor DOX, a common cytotoxic agent used in breast cancer treatment. We classified cancer cells according to their sensitivity to DOX and identified known and new molecular biomarkers for differential DOX effects (Figure 4l,m and **Table** **2**). For example, Isg15 protein is the product of the *Isg15* (IFN‐stimulated 15) gene and is a ubiquitin‐like protein that is induced by conditions of DNA damage.^[^ ^22^ ^]^ Previous studies propose Isg15 as a tumor suppressor due to its role in chemosensitivity of cancer via regulation of the p53 family member p63.^[^ ^23^ ^]^ Additionally, the genotoxic stress conditions induced by DNA‐damaging agents, such as DOX, causes Isg15 to conjugate with p53 to enhance its antitumor effects in vivo.^[^ ^24^ ^]^ This was observed in BALB/c nude mice treated with DOX, whereby Isg15 promotes the binding of p53 to itself and its target proteins, such as *Bax*, *Mdm*, and *Cdkn1* to suppress cancer cell proliferation.^[^ ^24^ ^]^ Another notable signature identified in the gene list was Bst‐2 or tetherin (encoded by *Bst2*), a transmembrane protein that has the paradoxical functions of providing innate immunity against viral infections and having protumorigenic functions. It has been used as a potential therapeutic target in the treatment of bladder cancer and endometrial cancer.^[^ ^25^ ^]^ According to the Human Protein Atlas, high BST‐2 expression level indicates a good prognosis in breast cancer. Congruent with this finding, a study by Legrier et al. reported that BST‐2 transcription is induced by IFN/STAT1 signaling in mice bearing breast cancer patient‐derived xenografts (PDXs) treated with chemotherapy at early timepoints. Activation of the IFN/STAT1 pathway in cancer cells as a result of treatments showed a positive correlation with response to chemotherapy.^[^ ^26^ ^]^ However, BST‐2 also appears to have opposing functions where its high expression is correlated with breast tumor aggressiveness, cancer cell survival, and metastasis.^[^ ^27^ ^]^ Finally, some of the other upregulated signatures identified in responders were also found to have an antitumorigenic association (Table 2). For example, tubulointerstitial nephritis antigen‐like 1 (*Tinagl1*) which has been found to suppress tumorigenesis and metastasis in triple‐negative breast cancer,^[^ ^28^ ^]^ and cystatin‐M (Cst6) a cysteine protease inhibitor that is epigenetically silenced during breast cancer progression and whose activity is associated with suppression of tumor malignancy.^[^ ^29^ ^]^

**Table 2:** Molecular markers for differential DOX effects in ALTEN‐engineered MMTV‐PyMT tumouroids

Gene Symbol	p_val	avg_logFC	pct.1	pct.2	p_val_adj	state	pct1‐pct2
Asns	0	0.9681	0.541	0.083	0	IInsens./Resist.	0.458
Stbd1	0	0.9018	0.568	0.132	0	IInsens./Resist.	0.436
P4ha2	1.44E‐295	0.8586	0.577	0.155	2.59E‐291	IInsens./Resist.	0.422
Trib3	1.10E‐300	0.9132	0.537	0.13	1.98E‐296	IInsens./Resist.	0.407
C1qbp	7.87E‐297	0.8740	0.731	0.327	1.41E‐292	IInsens./Resist.	0.404
Bnip3	0	1.0858	0.837	0.454	0	IInsens./Resist.	0.383
Cebpg	6.76E‐248	0.7288	0.589	0.208	1.21E‐243	IInsens./Resist.	0.381
Atf4	0	1.1161	0.874	0.499	0	IInsens./Resist.	0.375
Mthfd2	1.06E‐286	0.6727	0.424	0.056	1.89E‐282	IInsens./Resist.	0.368
Hspa9	0	1.2999	0.869	0.502	0	IInsens./Resist.	0.367
Angptl6	8.79E‐307	0.7932	0.397	0.033	1.58E‐302	IInsens./Resist.	0.364
Cyb5r1	2.34E‐262	0.7507	0.445	0.084	4.20E‐258	IInsens./Resist.	0.361
Slc2a1	0	1.2928	0.842	0.485	0	IInsens./Resist.	0.357
Syce2	7.68E‐239	0.7062	0.449	0.097	1.38E‐234	IInsens./Resist.	0.352
Leprotl1	1.54E‐218	0.7358	0.607	0.268	2.77E‐214	IInsens./Resist.	0.339
Jmjd6	2.18E‐209	0.7097	0.621	0.285	3.92E‐205	IInsens./Resist.	0.336
Rabggtb	1.92E‐197	0.6831	0.537	0.205	3.45E‐193	IInsens./Resist.	0.332
Gadd45a	2.41E‐227	1.0775	0.699	0.368	4.33E‐223	IInsens./Resist.	0.331
Hnrnpdl	1.41E‐195	0.7404	0.621	0.291	2.53E‐191	IInsens./Resist.	0.33
Gars	1.57E‐202	0.6373	0.468	0.14	2.83E‐198	IInsens./Resist.	0.328
Snrpa1	1.19E‐191	0.6414	0.588	0.261	2.13E‐187	IInsens./Resist.	0.327
Alkbh5	7.87E‐198	0.6447	0.683	0.357	1.41E‐193	IInsens./Resist.	0.326
Errfi1	1.73E‐190	1.9140	0.603	0.279	3.11E‐186	IInsens./Resist.	0.324
Tmem248	2.13E‐188	0.6293	0.588	0.265	3.83E‐184	IInsens./Resist.	0.323
Rad23b	5.34E‐168	0.5738	0.602	0.285	9.59E‐164	IInsens./Resist.	0.317
Sars	2.67E‐241	0.7232	0.762	0.447	4.79E‐237	IInsens./Resist.	0.315
Ero1l	0	1.7237	0.892	0.579	0	IInsens./Resist.	0.313
Nars	2.08E‐233	0.8093	0.72	0.414	3.73E‐229	IInsens./Resist.	0.306
Bri3	1.73E‐258	0.7677	0.851	0.547	3.10E‐254	IInsens./Resist.	0.304
Ifrd1	2.49E‐165	0.8691	0.657	0.355	4.47E‐161	IInsens./Resist.	0.302
Hist1h4i	0	1.9589	0.788	0.138	0	Response	0.65
Cst6	0	1.8902	0.815	0.194	0	Response	0.621
Slc4a11	0	1.2655	0.749	0.14	0	Response	0.609
Isg15	0	1.4895	0.729	0.148	0	Response	0.581
Bst2	0	1.9415	0.932	0.36	0	Response	0.572
Tinagl1	0	1.5670	0.88	0.34	0	Response	0.54
Ift27	0	1.2479	0.679	0.15	0	Response	0.529
Pmaip1	0	1.0826	0.592	0.063	0	Response	0.529
Ckmt1	0	1.3361	0.633	0.123	0	Response	0.51
Cirbp	0	1.5625	0.897	0.389	0	Response	0.508
Prcp	0	1.1002	0.621	0.116	0	Response	0.505
Dusp28	0	0.9930	0.589	0.088	0	Response	0.501
Irf7	0	1.3872	0.601	0.101	0	Response	0.5
Psapl1	0	1.0040	0.524	0.031	0	Response	0.493
Gm13056	0	1.1043	0.639	0.157	0	Response	0.482
Dcxr	0	1.3440	0.833	0.364	0	Response	0.469
Ly6a	0	2.3923	0.619	0.155	0	Response	0.464
Taf13	0	1.1167	0.779	0.321	0	Response	0.458
Glud1	0	1.0120	0.691	0.239	0	Response	0.452
Ccng1	0	1.0293	0.878	0.428	0	Response	0.45
Itm2a	0	1.2452	0.501	0.053	0	Response	0.448
Hist1h2ac	0	1.1188	0.494	0.055	0	Response	0.439
Ehd4	0	0.9410	0.63	0.191	0	Response	0.439
Ak1	0	0.9247	0.704	0.265	0	Response	0.439
Ecm1	0	1.4866	0.707	0.272	0	Response	0.435
Htra1	0	1.2385	0.515	0.082	0	Response	0.433
Gpaa1	0	0.8693	0.615	0.183	0	Response	0.432
Dhrs1	0	0.9890	0.598	0.174	0	Response	0.424
Fuca1	0	1.1154	0.81	0.387	0	Response	0.423
Tmem238	0	0.9939	0.771	0.351	0	Response	0.42

Within the molecular pathways of DOX insensitivity or resistance, we identified for example high expression of *Asns* and *Trib3*. *Asns*, encodes by asparagine synthetase, a protein known to induce DOX resistance in uterine cancer^[^ ^30^ ^]^ and ALL^[^ ^31^ ^]^ and has been proposed as a predictive biomarker for ovarian cancer and a therapeutic target for non‐Hodgkin's lymphoma.^[^ ^32^ ^]^ Similarly, the pseudokinase TRIB3 expression is induced in response to cellular stress^[^ ^33^ ^]^ and protects against the apoptotic effects of DOX in gastric cancer.^[^ ^34^ ^]^ TRIB3 abolished the cytotoxic effects of the mTOR inhibitor Rapalogs, preventing spliceosome dysregulation^[^ ^35^ ^]^ and thus placing TRIB3 as a potential biomarker to predict drug insensitivities to multiple drugs used in anticancer therapy. These examples illustrate the potential of ALTEN to identify the molecular pharmacological mechanisms and multidrug resistance, one of the current grand challenges of cancer therapy.^[^ ^30^ ^]^

Cancer immunotherapy, especially checkpoint blockade, revolutionized the landscape of cancer treatment and significantly increased patient survival in a range of cancers.^[^ ^36^ ^]^ However, a major challenge to the study of the effects of drugs that target the immune system is that current conventional preclinical models—cocultures or PDXs—cannot to recapitulate the complex immune lineages and cellular niches of the tumor microenvironment, thus limiting the analysis of mechanistic effects of immunotherapy. Here, as a proof of concept, we have used ALTEN to culture clinical specimens of precancerous gastric tissue and a breast cancer tumor and study T cell responses to the immunomodulator IL2, ex vivo and within the tumor microenvironment at the single cell level. IL‐2 is a pleiotropic cytokine that plays a critical role in the immune response.^[^ ^37^ ^]^ High dosage of IL‐2 treatment is one of the first successful immunotherapy to metastatic cancers.^[^ ^38^ ^]^ We found IL‐2 promotes tumor‐infiltrating CD8+ T‐cell responses in ALTEN preserved tissues, recapitulating the observation from a recent tumor xenograft model.^[^ ^18^ ^]^ To our knowledge, ALTEN is the first rapid and cost‐effective method to assay the effects of immunomodulators ex vivo in preserved cellular niches.

We envision ALTEN to be a highly useful research tool for the multidimensional analysis of drug responses to tissues in situ to a wide range of chemical perturbations, including immunomodulation of tumor‐infiltrating immune cells. To enable high content‐screening standardization of applications, we designed a scalable device for automation and miniaturization of the ALTEN encapsulation process. The ALTEN‐device produces a homogeneous, reproducible, and robust output with fine control of the hydrogel size. The automated version of ALTEN together with the utility of ALTEN as a preservation method for tissue transport makes an ideal platform to ensure consistency in large‐scale multicenter endeavors.

In summary, we developed ALTEN, an ex vivo tissue preservation platform, that acts as an intermediate matrix between 3D spheroids and in vivo drug testing models—more efficient and practical for the investigation of molecular pharmacological mechanisms and early drug discovery that could be applied to multiple diseases. ALTEN enables a systematic and standardized capacity to study high‐resolution molecular responses among heterogeneous cell populations within original niches. We also demonstrate the full compatibility of this tissue engineering technique with single cell resolution genomic applications, paving the way for the application of this technology for accurate target validation.

## 4 Experimental Section

### Cell Culture

The mCherry‐expressing mammary adenocarcinoma cell lines 4T1.2 and 67NR (generously provided by Prof. Robin Anderson) were cultured in Minimum Essential Medium Alpha with 5% v/v fetal bovine serum (FBS). Cell lines were tested for mycoplasma and were cultured in 37 °C at 5% CO_2_. Cells were passaged when confluence reached 80%.

### Experiments Involving Animals and Human‐Derived Tissues

All animal procedures and maintenance of mice were conducted in accordance with the Garvan Institute of Medical Research/St. Vincent's Hospital Animal Ethics Committee (AEC)—animal ethics number 19/02. Mice were provided with food and water ad libitum. The collection and analysis of human tissues were conducted according to the Human Research Ethics Committee, from HREC X13‐0133, Royal Prince Alfred Hospital, Sydney Local Health District and 17/176 HREC/17/POWH/389, Prince of Wales Hospital, South East Sydney Local Health District, and from the Institutional Review Board of Stanford University (IRB 44036 and IRB 45077).

### Development of Tumor Bearing Mice

For the implantable mammary tumor models, 8‐week‐old female BALB/c mice (ABR, Mossvale, New South Wales, Australia) were intraductally injected with 67NR (2 × 10^5^) or 4T1.2 (5 × 10^5^) tumor cells in the fourth inguinal mammary glands, under general anesthesia (Induction: 1L min^−1^ oxygen with 4% Isoflurane; Maintenance: 1L min^−1^ oxygen with 2% Isoflurane). The nipple of one side of the fourth inguinal mammary gland was removed using spring scissors and cells were injected via a Hamilton syringe with a 30‐gauge blunt‐ended fixed 1/2‐inch needle. Animals were placed in a half on/half off recovery box over a heat pad to prevent hypothermia after surgery. Animals were consistently monitored weekly post‐surgery until tumors were collected. The MMTV‐PyMT animals were used as a spontaneous primary mammary tumor model. At 12–14 weeks old, tumors were collected from all mammary glands.

### Tissue Encapsulation in Alginate and Treatments

Tissues were cut into 1 mm^3^ size explants using surgical scissors and were encapsulated into 20 µL of alginate droplets on top of a hydrophobic surface. The droplets were transferred to a cross‐linking agent, 0.1 m calcium chloride solution, for 10 min to allow the formation of the alginate beads. Excess CaCl_2_ was then washed off with DMEM media. Beads were cultured individually in a 48 well plate at 37 °C in 5% CO_2_ with DMEM media supplemented with 10% FBS, 72U insulin, 2 mm glutamine, 10 mm HEPES, 10 ng mL^−1^ EGF, and 10 ng mL^−1^ cholera toxin. Tissues were treated with DOX (Selleckchem S1208) to a final concentration of 2 µm. The media was changed every 2nd day.

Sodium alginate (NovaMatrix, Sandvika, Norway) was prepared in dH_2_O at a concentration of 1% w/v and dissolved overnight at 37 °C in a rotary suspension mixer. The alginate solution was then filter sterilized with a 0.22 µm filter.

Alginate beads were collected and subjected to 55 mm sodium citrate in 0.1 m EDTA for 10 min to dissolve the alginate. The recovered tissue was then washed in phosphate buffer saline (PBS) with 2% FBS. The wash solution was discarded, and the tissue was used for downstream processes.

### Tissue Digestion and Flow Cytometry

Tissues were digested enzymatically with 15 000U collagenase (Sigma C9891) and 5000U hyaluronidase (Sigma H3506) in DMEM media at 37 °C for 1 h at 200 rpm within a shaking incubator. The samples were then further digested in 2.5% trypsin with 1 mm EGTA in PBS for 1min at 37 °C. Red blood cells were lysed with 155 mm ammonium chloride for 3 min at 37 °C and the sample was filtered through a 40 µm filter. A detailed protocol has been previously described.^[^ ^39^ ^]^ Flow cytometry was performed using the BD FACS Symphony for analysis and FACS Aria III for sorting. The following antibodies were used for flow cytometry EpCAM (Clone G8.8), CD45 (Clone 30‐F11), CD11b (Clone M1/70), F4/80 (Clone BM8), Ly6C (Clone HK1.4), Ly6G (Clone 1A8), CD3 (Clone 17A2), CD4 (Clone GK1.5), CD8 (Clone 53‐6.7), and B220 (Clone RA3‐6B2). Cell proliferation was monitored with CellTrace Cell Proliferation Kit following the manufacturer's protocol. Flow cytometry data were analyzed using the software package FlowJo (version 10.4.2). PyMT tumors used for single cell RNA‐sequencing were processed following the 10× Genomics Chromium Single Cell Protocol (V2 Chemistry, CG00052).

### Patient Biopsy Collection and Processing

Fresh whole tumor tissue was dissected from mastectomy specimens by a Pathologist, within 30 min of resection from St. George Hospital and transported to the Garvan Institute of Medical Research in DMEM media supplemented with 10% FBS. Biopsies were cut into 1 mm^3^ using a Surgical Scalpel Blade No. 22 (Swann‐Mortann, SP, UK, Cat# 0208). Tumor pieces were encapsulated in alginate following the methodology as described previously. Tissues were allocated to either control or IL2 treated group, where IL‐2 (BioLegend, CA, USA, Cat# 589104) was added to a final concentration of 100 ng mL^−1^.

Biopsies were digested following a modified protocol of the MACS Human Tumor Dissociation Kit (Miltenyi Biotech, NSW, Australia, Cat #130‐095‐929). The biopsy samples were released from alginate encapsulation and were subsequently washed with RPMI 1640 medium (Gibco/Life Technologies; VIC, Australia, Cat # 11875‐119). The digestion enzyme mix was prepared accordingly to the protocol of the MACS Human Tumor Dissociation Kit, where it was then incubated with the biopsy samples at 37°C for 40min at 200rpm within a shaking incubator. Each sample was individually disrupted with a P1000 Gilson Pipette at 10min interval during the incubation. Samples were washed with PBS supplemented with 2% FBS and centrifuged at 1200 rpm for 5 minutes at 4°C. The pellet was then resuspended with 2% FBS in PBS for use in flow cytometry analysis or sorting.

For FACS analysis, cell suspensions were incubated with anti‐human antibodies for EpCAM (Clone 9C4, BioLegend, Cat # 324205) and CD45 (Clone HI30, BD Bioscience, Cat# 560566) on ice in the dark for 30 min before they were washed, centrifuged, and resuspended with 2% FBS in PBS for analysis using the BD FACS Symphony.

To allow multiplexing in a run of 10× Genomics Chromium, cell hashing with barcode antibodies were used for the breast tumor patient samples (vehicle and IL2) as previously described.^[^ ^40^ ^]^ Cell suspension was resuspended in one of two hashtag solution, vehicle samples with TotalSeq‐A0251 anti‐human Hashtag 1 Antibody (GTCAACTCTTTAGCG, Biolegend, Cat# 394601) and IL‐2 treated samples with TotalSeq‐A0252 anti‐human Hashtag 2 Antibody (TGATGGCCTATTGGG, Biolegend, Cat# 394603) diluted to a final concentration of 10 µg mL^−1^ in 2% FBS in PBS. Samples were incubated on ice in the dark for 30 min before they were washed, centrifuged, and resuspended with 2% FBS in PBS for sorting with BD FACSAria III Cell Sorter for subsequent single cell capture.

### Electroforce Analysis

Fresh, ALTEN encapsulated tumor, and noncapsulated MMTV‐PyMT tumor tissues (*N* = 5 each) were used for the measurement of mechanical properties. Both encapsulated and noncapsulated tumors were cultured ex vivo for 24 h. Tissue samples (width = 10 mm and length ≈ 20 mm) were dissected using a feather blade microtome and their thicknesses were measured using a digital caliper. The final thickness for each sample was an average of four measurements. Sample preparation for mechanical testing was explained before in previously published papers. Briefly, a 5 mm length in the middle of the sample was identified and sandpapers (250 grit) were bonded above and below the sample on each edge using a cyanoacrylate adhesive. Samples were initially equilibrated in 0.15 m PBS at room temperature for 5 min. An ElectroForce testing machine (Biodynamic 5100, TA Instruments, USA) was employed to conduct mechanical tests. Mechanical characterizations (displacement control) were performed at the strain rate of 0.05 mm s^−1^ up to 60% strain to be nondestructive, yet high enough to include the linear region. Engineering stress–strain curves that were prepared using a 4th‐degree polynomial fit function were used to characterize the mechanical properties. Outcome measures of Young's and toe moduli, maximum stress, and resilience were calculated using Matlab (R2021a, The Mathworks Inc.). Maximum stress was defined as the peak stress that was recorded during the test. Modulus was calculated as the slope of the best‐fit line to the stress–strain curve with toe modulus representing the initial region of the mechanical test. Resilience, the ability of the tissue to absorb energy in the elastic region, was the area under the stress–strain curve.

### Immunohistochemistry, Immunofluorescence, and Microscopy

Tumor and lungs were fixed in 10% neutral buffered formalin overnight. Formalin‐fixed paraffin embedded (FFPE) samples were cut into 4 µm sections for immunohistochemical staining. Slides were deparaffinized before staining for H&E, Ki67, CC3, and Picrosirius red. Ki67 and CC3 staining were performed on a Leica Bond RX (Leica Biosystems, Wetzlar, Germany) using the Ki67 antibody (Thermo Scientific, NSW, Australia, Cat# RM‐9106‐S1) and CC3 antibody (Cell Signaling, MA, USA, Cat# 9661) at 1:500 dilution, following the manufacturer's Bond Polymer Refine IHC protocol (no haematoxylin) with a 60 min antibody labeling time. Slides were then counterstained with haematoxylin using the Shandon Instant Haematoxylin Kit and cover slipped. H&E staining were conducted with a standardized protocol and cover slipped. Slides stained for Picrosirius red were first stained in haematoxylin for 30 s before treatment with 0.2% w/v aqueous phosphomolybdic acid for 2 min at room temperature. Direct Red 80 was dissolved in saturated picric acid solution to produce the Picrosirius red solution, which was used to stain the slides for 1 h. Acidified water made from 0.5% acetic acid in distilled water was used to wash excess Picrosirius red solution off the slides for 2 min with and slides were then submerged in 70% ethanol for 1 min. The slides were dehydrated, and cover slipped. Sections were photographed with a Leica DM4000 light microscope under an objective lens of 5× or 10×. All images were quantified using Andy's Algorithm, a series of automated image analysis pipelines in FIJI.^[^ ^41^ ^]^

For immunofluorescence analyses, FFPE sections (4 µm) were dewaxed and rehydrated through xylene and graduated ethanol, and antigen retrieval was performed by boiling slides in a pressure cooker in citrate buffer (0.01 m citric acid in deionized water, pH 6.0). Labeling with primary antibodies was performed in blocking buffer of 5% normal goat serum prepared in CAS‐Block (Thermo Fisher Scientific). Antibodies used were anti‐Fibronectin (BD Biosciences, Cat# 610077, 1/100), anti‐Periostin (Sigma‐Aldrich, Cat# SAB4200197, 1/500), and anti‐Tenascin C (Merck Millipore, Cat# AB19013, 1/100). AlexaFluor555‐ or AlexaFluor488‐conjugated secondary antibodies (Thermo Fisher Scientific, 1/400) were used to detect positive signals. Sections were counterstained with anti‐ECadherin‐Alexa647 (BD Biosciences, Cat# 560062, 1/200) and DAPI (Thermo Fisher Scientific) before mounting with ProSciTech mounting medium. Images were acquired using a Zeiss LSM700 confocal system.

Multiphoton microscopy was conducted using a Leica DMI 6000 SP8 Confocal at 25× magnification to image collagen deposition and tissue structure of ALTEN‐engineered tissues using second harmonic generation (SHG). SHG/collagen signals were collected using an excitation wavelength of 920 nm with a detector of HyD7 460 nm cube; mCherry was excited at 561 nm wavelength and detected with a HyD4 607–647 nm cube; GFP was excited at 488 nm wavelength and detected with a HyD4 510 nm cube. Bright‐field transmission images were co‐acquired with SHG data.

Cell viability was assessed using the Calcein‐AM and propidium iodide staining kit (SIGMA) dissolved in fresh medium, 1:500 for Calcein‐AM and 1:1000 for propidium iodide and following manufacturer's instructions. Fluorescence was assessed under a microscope: Calcein‐AM excitation: 494 nm, emission: 517 nm and propidium iodide excitation: 536 nm, emission: 617 nm.

### Statistical Analysis

Statistical analysis of flow cytometry and imaging data was performed using the GraphPad Prism 9 (GraphPad Software, San Diego, USA). The statistical test used in each analysis is explained in the figure legends. In brief, statistical significance was defined as *p*‐value <0.05. To compare two groups, Student *t*‐test was carried out. For comparisons of multiple groups, one‐way ANOVA testing followed by Dunnett's multiple comparison test, with a single pooled variance, was used instead. The *p*‐value of the cell viability assays using Calcein‐AM and propidium iodide was calculated using a two‐tailed *t*‐test with Origin 8.0.

For statistical analysis of the electroforce experiments, all data were first assessed for normality using the Shapiro–Wilk test. To identify the statistical differences for the mechanical parameters, separate one‐way ANOVA tests were conducted for each variable of Young's modulus, toe modulus, maximum stress, and resilience having a fixed factor of sample groups (fresh tissue, fresh tumor, encapsulated tumor, and noncapsulated tumor) using an alpha of 0.05. Posthoc multiple comparisons were conducted using a Bonferroni adjustment on alpha. This statistical analysis was performed using Matlab (R2021a, The Mathworks Inc.).

### Bioinformatic Analysis of Single Cell Data

The Cell Ranger pipeline (version 3.0.2) was used for alignment to genome mm10 or hg38, filtering, barcode counting, and UMI counting from FASTQ files. Doublets in each sample were removed using *DoubletFinder*.^[^ ^42^ ^]^ Downstream analysis was performed using the Seurat package (v3.0). To demultiplex human breast tumor samples stained with hashtag oligos, the authors used the *HTODemux* Seurat function and cells identified as singlets were extracted. *DoubletFinder* was not needed in samples stained with hashtag oligos as empirical doublets could be detected using Cell hashing.^[^ ^40^ ^]^ A summary of the number of cells metrics is shown in **Table** **3**. Data were normalized using *SCTransform* function^[^ ^43^ ^]^ and to remove batch effect between captures the datasets from different captures (between baseline, day 1 and day 3 captures or between Sydney and Melbourne capture) were integrated using the “anchor” method.^[^ ^11^ ^]^ UMAP plots of PyMT data were generated using the five‐sample object and after subsetting for culture analysis (baseline, day 1 vehicle and day 3 vehicle) and for DOX effect analysis (day 1 vehicle vs DOX and day 3 vehicle vs DOX) (Figure S5e, Supporting Information). The Single R package^[^ ^12a^ ^]^ and cell markers were used to assign cell identities to the unsupervised cell clusters using the *ImmGenData* ^[^ ^12b^ ^]^ and *HumanPrimaryCellAtlas*.^[^ ^44^ ^]^ The DOX response gene signature used was obtained from ref. [14] (see Table 1 for full list). Unsupervised single‐cell pseudotime trajectories were performed on the epithelial‐labeled cells of the PyMT data using the Monocle package (v2.0).^[^ ^45^ ^]^ Cells were ordered based on differential genes between the vehicle and DOX conditions using the *differentialGeneTest* function. GSVA^[^ ^46^ ^]^ was calculated for averaged expression values for clusters or Gaussian distributed counts data using mouse or human C2 curated gene sets relevant for DOX or IL2 treatment downloaded from http://bioinf.wehi.edu.au/software/MSigDB.

**Table 3:** Summary of scRNAseq metrics and quality control filters

Dataset	Total reads per UMI used	Median nUMI per cell	Median nGene per cell	Total cells	Doublets	Low quality	N cells passing filter	Epithelial cells	T cells
PYMT_baseline	24445244	3869.5	1295	5494	214	232	5048	4266	NI
PYMT_ALTEN_day1_control	22912489	3304.5	1112.5	6044	278	292	5474	4932	NI
PYMT_ALTEN_day1_dox	18233734	3735.5	1281	4520	176	189	4155	3306	NI
PYMT_ALTEN_day3_control	23736029	6480.5	1998	3428	79	193	3156	2843	NI
PYMT_ALTEN_day3_dox	23216489	4682	1693	4430	137	241	4052	3777	NI
PYMT_ALTEN_Sydney	24445244	3869.5	1295	5494	214	295	4985	4367	NI
PYMT_ALTEN_Melbourne	86948068	9592	2510	6743	370	5	6373	5809	NI
BC_ALTEN_control^a)^	11468824	4839	1858	2523	130	173	2220	NI	2015
BC_ALTEN_IL2^a)^	15380904	5321	2055	3015	130	221	2664	NI	2388
GIM_ALTEN_control	13899809	2408	927	3331	78	499	2754	NI	573
GIM_ALTEN_IL2	13052758	2548	909	3124	70	553	2501	NI	321

NI=Not Investigated

^a)^ 260 doublets based on hashtags.

## Conflict of Interest

The authors declare no conflict of interest.

## Author Contributions

A.M.K.L., J.C., and Y.C.‐S. contributed equally to this work. H.P.J. and D.G.‐O. are co‐senior authors. Conceptual and experimental design: A.M.K.L., J.C., H.P.J., and D.G.‐O. Data acquisition and experimentation: A.M.K.L., J.C., H.L., G.F., S.T.B., L.R.d.l.F., L.C., J.S., L.M., J.L.T., and F.V.‐M. Data analysis: A.M.K.L., J.C., Y.C.‐S., S.M.G., H.L., G.F., S.T.B., L.R.d.l.F., J.V., F.J.R., T.R.C., L.M., F.V.‐M., and D.G.‐O. Provided reagents and expertise: R.J.H., E.K.A.M., J.B., M.S.S., T.R.C., J.T. (Tipper), C.J.O., L.M., D.J., F.V.‐M., H.P.J., and D.G.‐O. Bioinformatic Analysis: J.C., Y.C.‐S., S.M.G., J.V., F.J.R., and D.G.‐O. Data visualization: J.C., Y.C.‐S., S.M.G., J.V., and D.G.‐O. Data interpretation: A.M.K.L., J.C., Y.C.‐S., L.M., F.V.‐M., H.P.J., and D.G.‐O. Manuscript writing: A.M.K.L., J.C., Y.C.‐S., F.V.‐M., H.P.J., and D.G.‐O. All authors read and approved the final manuscript.

## Supporting information

Supporting Information

## Data Availability

The data that support the findings of this study are openly available in NIH, National Library of Medicine. National Centre for Biotechnology Information at https://dataview.ncbi.nlm.nih.gov/objects?linked_to_id=PRJNA678538&archive=sra, reference number 678538.
