# Supplementary material for: ALTEN: A High‐Fidelity Primary Tissue‐Engineering Platform to Assess Cellular Responses Ex Vivo
Source: Adv Sci (Weinh). 2022 May 25;9(21):2103332. doi: 10.1002/advs.202103332 (PMC9313544; doi:10.1002/advs.202103332)
Supplement: Supplementary file 1 — Supporting Information [file ADVS-9-2103332-s001.pdf]

ADVANCED SCIENCE
Open Access

Supporting Information

for *Adv. Sci.*, DOI 10.1002/advs.202103332

ALTEN: A High-Fidelity Primary Tissue-Engineering Platform to Assess Cellular Responses Ex Vivo

*Andrew M. K. Law, Jiamin Chen, Yolanda Colino-Sanguino, Laura Rodriguez de la Fuente, Guocheng Fang, Susan M. Grimes, Hongxu Lu, Robert J. Huang, Sarah T. Boyle, Jeron Venhuizen, Lesley Castillo, Javad Tavakoli, Joanna N. Skhinas, Ewan K. A. Millar, Julia Beretov, Fernando J. Rossello, Joanne L. Tipper, Christopher J. Ormandy, Michael S. Samuel, Thomas R. Cox, Luciano Martelotto, Dayong Jin, Fatima Valdes-Mora, Hanlee P. Ji and David Gallego-Ortega**

Supplementary text:

Selection of scRNAseq data integration approach.

We performed scRNAseq analysis of the fresh PyMT tumour (baseline), and the same tumour sample after ALTEN-culturing for 1 day or 3 days with the presence of DOX or the vehicle control. Due to the design of this experiment, we had 3 captures performed over 3 different days: baseline-batch 1, ALTEN day 1 - batch 2 and ALTEN day 3 – batch 3. The vehicle or DOX treated samples from the same day were capture on the same 10X Chromium chip using half volumes of the same reagents to minimise batch effect. In fact, when we merged the data of each condition without integration we observed a batch effect (Figure S5a) and it was more pronounced between captures performed in different days than between samples run on the same 10X chip (Figure S5a-c), as previously reported (*Chromium 10X Technical Note: Biological & Technical Variation in Single Cell Gene Expression Experiments (Document CG00055*). However, there is a significant transcriptional difference between Vehicle and DOX samples from day 3 (Figure S5c), driven by the transcriptional differences of the epithelial cells (Figure S5b). To determine if the transcriptional shift between the Vehicle and DOX treated samples is caused by technical variation or biological changes driven by DOX treatment, we perform integration using two approaches, either per batch or per sample using two integration methods developed to correct batch effects, "anchor integration" from Seurat V3[1] or Reciprocal PCA (RPCA) integration from Seurat V4 (Figure S5e-l). Before integration, we find upregulation of a DOX response gene signature previously reported[2] (Figure S5d), as well as two new DOX response marker genes Isg15 and Bst2 (Figure S5d) that we have validated by IF (Figure 4m). However, after data integration the upregulation of the DOX signature and the Isg15 and Bst2 after 3 days of doxorubicin treatment was reduced or completely erased if the correction was done per sample (Figure S5n,p,r,t). This data suggests that integration per sample is overcorrecting the data as validated gene expression changes caused by DOX treatment are completely abolished, and therefore we conclude that integration of captures performed on different days only is the best batch correction strategy. In addition, we see similar trends between the Seurat V3 and Seurat V4 integration, but we decide to use the integration method from Seurat V3 as it has been recommended in a recent benchmark of batch effect correction methods[3].

Figure legends to supplementary material:

Supp. Fig. 1. Extended information on mammary cancer tumouroids. a, Representative images of multiphoton microscopy of ALTEN-engineered 4T1.2 tumouroids over the time. Red = cancer cells (mCherry), blue = second harmonic generation (SHG) signal (collagen fibres). **b**, Percentage of cell viability assayed by DAPI in the 4T1 samples cultured in ALTEN shown in Figure 1e. Data are represented as mean ± SEM **c**, Head-to-head comparison of the percentage of cell viability, assayed by flow cytometry (DAPI), of cell suspensions produced by equivalent volumes of ALTEN-engineered MMTV-PyMT tumouroids and two replicates of Air-to-Liquid Interphase (ALI) organoids after 7 days in culture compared with the fresh tissue prior to culturing. **d**, Flow cytometry gating strategy and representative comparison of the 4T1.2 cell suspensions from fresh tissue (baseline) vs. ALTEN-cultured 4T1.2 tumoroids for 3 and 7 days **e**, Cell live tracker intensity on mCherry+ cancer cells over time, reduction of live tracker intensity represents a doubling cell event. **f**, Mechanical stress analysis of large (>10mm) fragments of MMTV-PyMT mammary tumours at baseline (fresh tissue) or cultured for 24h in ALTEN or un-encapsulated. Left panel, average stress-strain curves, for 60% strain, during tensile loading. Right panels, calculated toe and Young's moduli, resilience and maximum stress. Data are represented as mean ± SEM. P-values are calculated using one-way ANOVA testing followed by Bonferroni correction. ** $P < 0.01$, *** $P < 0.001$.

Supp. Fig. 2. a, Representative immunofluorescence image of Calcein AM (green) highlighting live cells and propidium iodide (PI, red) revealing dead cells of fresh PyMT-derived tumours and after 1 or 3 days of cultured in ALTEN, ALI or without encapsulation. **b.** Quantification of the calcein and PI fluorescence intensity. Data are represented as mean ± SD. N => 3. *P*-values are calculated using two-tailed *t*-test.* *P* < 0.05, ** *P* < 0.01, *** *P* < 0.001. **c,** Percentage of epithelial, stroma or immune measured by flow cytometry of fresh PyMT-derived tumours (baseline, B) and after ALTEN or ALI encapsulated culture for 1 and 3 days. Data are represented as mean ± SEM. P-values are calculated using one-way ANOVA testing followed by Dunnett's multiple comparison test. * *P* < 0.05, ** *P* < 0.01, *** *P* < 0.001, **** *P* < 0.0001.

Supp. Fig. 3. ALTEN preserves tissue architecture and viability of 67NR mammary carcinomas. a, Live cell number and percentage of cell viability assayed by FACS (DAPI) from cell suspension of 67NR mammary carcinomas cultured in ALTEN or in the fresh (baseline, time = 0) tissue. **b**, Number of live cells obtained on the tissues assayed in panel A divided by major lineages (67NR cancer cells are defined by expression of mCherry, immune cells are defined by CD45 and stromal cells are defined as double negative). **c**, Representative histology images of ALTEN-engineered 67NR tumouroids stained by H&E, picrosirius red (collagen) or immunolabelled with cleaved caspase 3 (CC3) and Ki67 antibodies. **d- f**, Quantification of the area stained in the histology images in panel **c** (n = 6). **g**. Representative images of multiphoton microscopy of ALTEN-engineered 67NR tumouroids over the time. Red = cancer cells (mCherry), blue = second harmonic generation (SHG) signal (collagen fibres). Data are represented as mean ± SEM P-values are calculated using one-way ANOVA testing followed by Dunnett's multiple comparison test, ns = *P* > 0.05, * *P* <0.05, ** *P* < 0.01, *** *P* < 0.001, **** *P* < 0.0001

Supp. Fig. 4. Flexibility of ALTEN culturing several tissues of diverse origin. a, Flow cytometry analysis of normal mammary gland tissue cultured in ALTEN for 3 and 7 days showing preservation of luminal and basal epithelial cell lineages and immune cells, and **b**, their quantification. **c**, Percentage of viability, assayed by flow cytometry (DAPI), of cells suspensions from ALTEN-engineered breast cancer clinical biopsies cultured for 24h (left) n = 2. Major tumour cell lineages as defined as EpCAM+ (cancer cells), CD45+ (immune cells) and double negative (EpCAM-/CD45-) (stromal cells) (right). P1= patient 1, P2 = patient 2. **d**, Histology H&E images of different clinical biopsies engineered as ALTEN tumouroids and cultured for 1, 2 or 3 days compared with the baseline tissue, please note baseline and day 2 of the gastric intestinal metaplasia biopsy are missing due to unavailability of sample. (Scale bars 100μm).

Supp. Fig. 5. Intratumoural heterogeneity according to spatial location. Graphic representation of the tissue heterogeneity of 4T1.2 tumours and lungs assayed by flow cytometry comparing the fresh tissue (baseline) and after 3 days under ALTEN culture conditions. Cell compartments were defined by mCherry (cancer cells), CD45 (immune cells) and double negative (stromal cells). Immune populations were further defined by the expression of CD11b+, F4/80+ (macrophages); CD11b+, F4/80-, Ly6G+, Ly6Clow (PMN-MDSCs), CD11b+, F4/80-, Ly6G-, Ly6C+ (M-MDSC); CD3+, CD8+ (T cytotoxic cells) and CD3+, CD4+ (T helper cells).

Supp. Fig. 6. scRNAseq data integration. a, UMAP plot of merged scRNA-seq data without integration coloured by sample. **b,** principal component analysis (PCA) of pseudobulk scRNAseq separated by cell compartment: endothelial, epithelial, fibroblasts and immune cells. **c.** PCA of pseudobulk of the complete dataset of single-cell RNAseq. **d,** violin plot showing the normalised expression of the metagene signature for doxorubicin response, Isg15 and Bst2 gene expression in

each sample. UMAP projection of integrated data using different approaches: the anchor method from Seurat V3 to integrate the batches (baseline, day 1 and day 3) (**e**) or all samples separately (**f**); or the RPCA method from Seurat V4 to integrate the different batches (**g**) or the samples (**h**). **i- l,** UMAP plots corresponding to sections e- h, but split by sample. **m- p,** violin plot showing the corrected expression of the metagene signature for doxorubicin response using the different integration methods. **q- t,** violin plot showing the corrected expression of Isg15 and Bst2 genes using the different integration methods.

Supp. Fig. 7. Quality control of scRNA-seq data and summary of the bioinformatic workflow. a, Violin plot displaying number of genes (left) and percentage of mitochondrial (mito) genes (right) per cell in each condition. **b,** UMAP visualization of the percentage of mitochondrial genes per cell. **c,** Percentage of mitochondrial genes per cell in each condition after removing the mitochondrial rich cluster. **d,** UMAP projections of 23,032 cells coloured by time in culture, independently of treatment (vehicle or doxorubicin). **e,** UMAP projections coloured by time in cultured and split by treatment condition (vehicle or doxorubicin). **f,** Schematic summary of bioinformatic analysis workflow. Dox sensitivity analysis: 1) Integration of all five samples (baseline, day 1-vehicle, day 1-doxorubicin, day 3-vehicle, day 3- doxorubicin). Doublets in each sample were removed using *DoubletFinder* [4], then the data was normalized using *SCTransform* function (10.1186/s13059-019-1874-1) in Seurat and cells captured in different batches (baseline, day 1 and day 3) were integrated using Seurat's anchor strategy[1] as explain in Figure S5. Finally, before further analysis, the cluster rich in mitochondrial genes (>20% mito genes) was excluded. 2) To investigate the effects of culturing tumours in ALTEN overtime, we subset all cells from the baseline, day 1-vehicle and day 3-vehicle from the 5-sample object and performed a new dimensional reduction and reclustering. 3) To investigate the effect of Doxorubicin treatment on cancer cells cultured in ALTEN at each timepoint, we first extracted the vehicle controls and doxorubicin treated cells at day 1 and day 3 timepoints and subset the cells from the epithelial lineage. We also preformed dimensional reduction and reclustering of cells at day 1 and day 3 separately.

Supp. Fig 8. Single cell transcriptome analysis of ALTEN-tumoroids over time. a, Mosaic plot showing the percentage of cells in each unsupervised cluster in the baseline condition and after 1 day or 3 days of culture in ALTEN. The width of the bars is proportional to the number of cells belonging to each cluster. **b,** heatmap showing the normalised expression data of the top five differential markers found in each cell lineage. **c,** UMAP plot showing epithelial cells from the baseline condition and after 1 day or 3 days of culture in ALTEN after performing dimensional reduction and re-clustering. **d,** Dot plot showing percentage of expressing cells and average expression of top marker genes for epithelial subtypes (from Valdes-Mora et al. Cell Reports)[5]. Coloured boxes mark gene markers for each epithelial subtype. **e,** UMAP visualization of epithelial cells coloured by subtypes (left) and split by culture condition (right). **f,** Violin plot showing the normalized expression of the metagene signature of luminal progenitor, basal and alveolar cells in each epithelial subtype.

Supp. Fig. 9. Single cell transcriptome analysis of doxorubicin treatment in ALTEN-tumoroids for 24 hours. a, Schematic representation of the experimental workflow. **b**, UMAP projection of epithelial cells cultured in the ALTEN system for 24 hours with doxorubicin (Dox, 3,306 cells) or vehicle (Veh, 4,932 cells) normalised using *SCTransform* function from Seurat. **c**, UMAP visualization showing the metagene signature of doxorubicin response (see Table 1) overall (left) and split based on condition (right). **d**, Violin plot showing the normalized expression of the metagene signature of doxorubicin response in vehicle and doxorubicin treated cells. **e,** Dot plot showing the percentage of expressing cells and average expression of seven genes from the doxorubicin response signature in untreated (Veh) and treated (Dox) cells.

Supp. Fig. 10. a, Representative immunofluorescence image of ALTEN encapsulated MMTV-PyMT-derived tumoroids stained with Calcein AM (green) for live cells and propidium iodide (PI, red) for dead cells of ALTEN tumoroid after treatment with ABT-263 (2.56μM or 5.13μM), Doxorubicin (1.75μg or 3μg), Latrunculin A (0.5μM or 1μM) , S63845 (25μM or 50μM) or a vehicle control. **b.** Quantification of the Calcein (live) and PI (dead) fluorescence intensity in each condition. Data are represented as mean ± SD. *P*-values are calculated using two-tailed *t*-test. N = 3. *P*-values are calculated using * $P < 0.05$, ** $P < 0.01$, *** $P < 0.001$.

Supp. Fig. 11. Single cell transcriptome analysis of GIM treated with IL2. a, UMAP projections coloured based on k-nearest neighbour clustering and split by condition. **b**, Heatmap showing cell lineage markers in each cell type. **c**, Dot plot showing the comparison between the percentage of expressing cells and average expression of lineage markers found in each cell type from vehicle (yellow) and IL2 treated (red) cells. **d,** Dot plot showing the comparison between the percentage of expressing cells and average expression of marker genes for CD8+ subtypes (resident memory (Trm), effector memory (Tem) type 1, Tem type 2, Tem type 3, and cytotoxic) from control (yellow) and IL2 treated (red) cells.

Supp. Fig. 12. Effects of IL2 treatment on other cells types within the gastric microenvironment. a, Subset of plasma cells and UMAP visualization after dimensional reduction and re-clustering of control and IL2 treated cells. **b**, Subset of mast cells and UMAP visualization after dimensional reduction and re-clustering of control and IL2 treated cells. **c**, Subset of fibroblasts and UMAP visualization after dimensional reduction and re-clustering of control and IL2 treated cells. **d**, Heatmap showing the normalised expression of top differential genes per cluster in the mast cell population. **e**, Heatmap showing the normalised expression of top differential genes per cluster in the fibroblast population.

Supp. Fig. 13. Immunomodulation of breast tumour infiltrated T cells *ex vivo.* a, UMAP projections of 4,403 T cells coloured based on k-nearest neighbour clustering and split based on treatment (control, 2,015 cells and IL2 treated, 2,388 cells). b, Dot plot showing normalised expression of marker and activation genes for natural killer (NK) cells and different subsets of T cells (left panel). UMAP plot showing T cell subtypes and NK cells clusters based on the dot-plot markers. c, Line plot showing percent of cells in each cluster in response to IL2 treatment. d, UMAP plot re-clustering T CD8 cells highlighting the effects of IL2 treatment. e, Trajectory analysis of the T CD8+ cells based on differential analysis between the control and IL2 treated cells using the DDRTree method in Monocle2 coloured by states or (f) coloured by Seurat clusters. g, GSVA of T cell functional signatures.

Supp. Fig. 14. ALTEN-device encapsulation of healthy tissue. Long term (14 day) mammary and lung tissue encapsulated using the 200μm ALTEN microfluidic device, stained with Calcein AM highlighting live cells (green) and propidium iodide highlighting dead cells (red). Scale bars 200μm).

Supp. Fig. 15. ALTEN as a high-fidelity transport method for whole-tissue specimens. a, Quality metrics for the scRNA-seq analysis performed in the fresh sample (Syd = Sydney) and after transport (Mel = Melbourne). **b**. UMAP visualisation of the Syd and the Mel samples. **c**. Unsupervised k-nearest neighbour clustering and split based on geographical location for the tissue processing (Syd, 4,985 cells (baseline) and Mel, 6,379 cells). The right panel shows the relative contribution of each

cluster per sample. **d-e**. Annotation of cell lineages based on SingleR signatures and the relative contribution by cell lineage in each of the samples (Syd vs Mel). **f**, Analysis of the cell cycle signature in the fresh sample (Syd) and after transport (Mel).

References Supplementary Text.

[1] T. Stuart, A. Butler, P. Hoffman, C. Hafemeister, E. Papalexi, W. M. Mauck, 3rd, Y. Hao, M. Stoeckius, P. Smibert, R. Satija, *Cell* **2019**, *177* (7), 1888, https://doi.org/10.1016/j.cell.2019.05.031.

[2] B. Gyorffy, V. Serra, K. Jurchott, R. Abdul-Ghani, M. Garber, U. Stein, I. Petersen, H. Lage, M. Dietel, R. Schafer, *Oncogene* **2005**, *24* (51), 7542, https://doi.org/10.1038/sj.onc.1208908.

[3] H. T. N. Tran, K. S. Ang, M. Chevrier, X. Zhang, N. Y. S. Lee, M. Goh, J. Chen, *Genome Biol* **2020**, *21* (1), 12, https://doi.org/10.1186/s13059-019-1850-9.

[4] C. S. McGinnis, L. M. Murrow, Z. J. Gartner, *Cell Syst* **2019**, *8* (4), 329, https://doi.org/10.1016/j.cels.2019.03.003.

[5] F. Valdes-Mora, R. Salomon, B. S. Gloss, A. M. K. Law, J. Venhuizen, L. Castillo, K. J. Murphy, A. Magenau, M. Papanicolaou, L. Rodriguez de la Fuente, D. L. Roden, Y. Colino-Sanguino, Z. Kikhtyak, N. Farbehi, J. R. W. Conway, N. Sikta, S. R. Oakes, T. R. Cox, S. I. O'Donoghue, P. Timpson, C. J. Ormandy, D. Gallego-Ortega, *Cell Rep* **2021**, *35* (2), 108945, https://doi.org/10.1016/j.celrep.2021.108945.
